# A Systematic Review on Haptic Feedback in Medical Robotics: Technologies, Applications, Clinical Translation, and an Information-Oriented Perspective

**DOI:** 10.3390/s26154824

**Published:** 2026-07-30

**Authors:** Momen Abayazid

**Affiliations:** 1 Control and Instrumentation Engineering Department, King Fahd University of Petroleum and Minerals, P.O. Box 1816, Dhahran 31261, Saudi Arabia; momen.abayazid@kfupm.edu.sa; 2 Interdisciplinary Research Center for BioSystems and Machines, King Fahd University of Petroleum and Minerals, P.O. Box 1816, Dhahran 31261, Saudi Arabia

**Keywords:** haptics, medical robotics, force feedback, tactile perception, teleoperation, rehabilitation robotics, surgical robotics, human–robot interaction, prosthetics, surgical simulation

## Abstract

Haptic technology restores the sense of touch to robotic systems and has become increasingly important for safe and intuitive human–robot interaction in healthcare. Despite substantial advances over the past two decades, widespread clinical adoption remains limited, highlighting a persistent gap between laboratory research and real-world medical deployment. This review synthesizes research from robotics, human–computer interaction, neuroscience, and clinical medicine based on a systematic literature search conducted in IEEE Xplore, PubMed, and Scopus (2000–2025). The review adopts an information-centric perspective, focusing on the clinically relevant information conveyed through haptic feedback rather than force reproduction alone. The review examines tactile, kinesthetic, and hybrid feedback modalities; summarizes key principles of haptic rendering, stability, and control; and evaluates applications in surgical robotics, teleoperation, rehabilitation, prosthetics, and medical training. Evidence indicates that haptic feedback can improve performance, reduce excessive forces, and enhance situational awareness, although benefits remain task-dependent. Clinical translation continues to be constrained by sensing limitations, miniaturization challenges, stability requirements, human factors, and regulatory considerations. Current research is increasingly directed toward sensorless force estimation, artificial intelligence-assisted haptic rendering, wearable and soft haptic interfaces, and neurohaptic technologies, reflecting a shift toward task-oriented and information-centric feedback. Future progress will depend less on maximizing physical realism and more on delivering clinically meaningful information through stable, interpretable, and user-centered haptic systems. This review provides a roadmap for advancing clinically deployable haptic technologies in healthcare.

## 1. Introduction

Human interaction with the physical world is fundamentally mediated by touch, the only sense that directly conveys mechanical interaction. Through tactile and kinesthetic cues, we perceive force, vibration, texture, stiffness, and motion capabilities essential for dexterous manipulation in medicine. In robot-assisted surgery and other medical robotic interventions, however, this natural sensory connection is disrupted. Surgical tools act as mechanical intermediaries that attenuate or filter tactile information, forcing clinicians to rely primarily on visual feedback. This sensory deprivation has measurable clinical consequences: studies show that operators using vision alone apply up to 50% more force than necessary, increasing tissue stress and potential micro-damage [1,2].

Haptic technology, derived from the Greek haptikos (“able to touch”), addresses this gap by artificially reproducing the sense of touch through mechanical, electrical, or vibrotactile stimulation. By restoring tactile and force feedback to medical robotic systems, haptics enables clinicians to perceive tissue properties, instrument interactions, and environmental constraints that visual feedback alone cannot convey. This sensory restoration is not merely ergonomic; it directly impacts patient safety, surgical precision, rehabilitation outcomes, and procedural efficiency [3,4].

### 1.1. The Cognitive Dimension of Touch

Beyond physical contact, touch contributes to situational awareness and cognitive efficiency. According to multiple-resource theory, human attention divides across sensory channels; off-loading mechanical perception to the tactile domain frees visual attention for spatial navigation and planning. Thus, adding haptic cues can reduce cognitive load and improve motor learning. In simulation environments, trainees who receive force feedback demonstrate faster skill acquisition and higher retention [3]. Haptic realism also enhances engagement, an important factor in telepresence and rehabilitation adherence.

### 1.2. Rationale and Scope

Despite two decades of research and development in medical haptics, clinical adoption remains limited. Most commercially available robotic surgical systems operate without true force reflection, and many rehabilitation robots provide only basic guidance forces. This gap between laboratory demonstrations and clinical implementation stems from coupled challenges in hardware design, control stability, human factors, and regulatory approval. While several reviews have addressed specific aspects of medical haptics (e.g., surgical applications or control methods), a comprehensive synthesis spanning physiological foundations, technological implementations, clinical applications, and translational barriers is lacking.

This work presents a systematic review with qualitative thematic synthesis rather than a quantitative meta-analysis. The review is based on a systematic literature search and structured study selection process to synthesize current evidence on haptic feedback in medical robotics. This review aims to provide such a comprehensive overview, with three primary objectives:

1. To systematically review the physiological basis of haptic perception and its engineering implementation in medical robotic systems
2. To analyze current applications across surgical robotics, rehabilitation, prosthetics, and training, highlighting both successes and limitations
3. To identify key technical and translational challenges and propose research priorities for advancing clinical adoption

#### Contribution

While prior reviews of medical haptics have often focused on specific application domains (e.g., surgery, rehabilitation, or prosthetics) or on individual technical topics such as force feedback and control stability, this review adopts a complementary information-centric perspective. The central distinction is between the physical capabilities of haptic hardware and the information conveyed to the user: what interaction variables are communicated, how they are encoded, and why they are clinically relevant.

Building on this perspective, the review examines haptic systems across surgical, rehabilitation, prosthetic, and simulation applications through a common framework encompassing information flow, rendering and guidance strategies, control-theoretic constraints (e.g., stability, passivity, and delay), human factors (e.g., perception, workload, and comfort), and clinical translation challenges. This approach highlights recurring design trade-offs that span application domains and emphasizes that clinically effective haptic systems often depend as much on appropriate information design as on force-rendering fidelity.

Unlike application-specific surveys, the objective is not to review individual medical applications in isolation but to identify common principles that govern the design, evaluation, and deployment of haptic systems in healthcare. By framing haptics as an information-delivery problem rather than solely a force-reproduction problem, the review provides a unified perspective for comparing tactile, kinesthetic, and hybrid feedback modalities and for evaluating haptic systems using clinically meaningful outcomes such as safety, targeting accuracy, workload, and usability.

The information-centric perspective proposed here therefore provides a common framework for analyzing these challenges and identifying opportunities for future development. From this perspective, the value of a haptic feedback channel is determined not solely by its physical fidelity, but by the extent to which it conveys information that improves task execution and clinical decision making. In practical terms, effective haptic information may be evaluated through task-level outcomes such as force safety margins, anatomical targeting accuracy, error recovery performance, workload reduction, fatigue mitigation, and overall procedural efficiency. Conversely, redundant information can be viewed as feedback that does not measurably improve task performance, falls below relevant perceptual thresholds, duplicates information already available through other sensory channels, or increases cognitive workload without improving clinical outcomes. Although a universally accepted quantitative framework for evaluating haptic information content does not yet exist, these criteria provide a practical basis for comparing alternative feedback modalities and guiding the design of clinically meaningful haptic systems. This perspective also motivates a shift from device-centered evaluation metrics alone toward outcome-oriented assessment that emphasizes safety, usability, and task performance in realistic clinical scenarios.

### 1.3. Research Questions

To structure the review and guide the literature synthesis, the following research questions are addressed:

RQ1: How are tactile, kinesthetic, and hybrid haptic technologies implemented in medical robotic systems?

RQ2: What rendering and control strategies enable safe and effective haptic interaction under clinical constraints?

RQ3: What evidence exists regarding the benefits of haptic feedback in surgical robotics, rehabilitation, prosthetics, and training systems?

RQ4: What technical, human-factor, and regulatory barriers currently limit widespread clinical adoption of medical haptics?

RQ5: How does an information-centric perspective help guide the future design and evaluation of haptic systems for healthcare applications?

### 1.4. Literature Search Methodology

To capture the breadth of the field while maintaining focus on medical robotic applications, seven query groups were developed around the principal domains of medical haptics. The search domains and representative query formulations are summarized in Table 1. These domains covered medical haptics, force feedback in robotic surgery, tactile perception, rehabilitation robotics, teleoperation, prosthetics, and surgical simulation. This systematic review was conducted and reported in accordance with the Preferred Reporting Items for Systematic Reviews and Meta-Analyses (PRISMA 2020) and it can be found in the Supplementary Material of this paper. The literature search, study selection, and reporting process followed the PRISMA 2020 guidelines. Because the objective was qualitative thematic synthesis rather than quantitative meta-analysis, the evidence was synthesized narratively.

**Figure 1:**
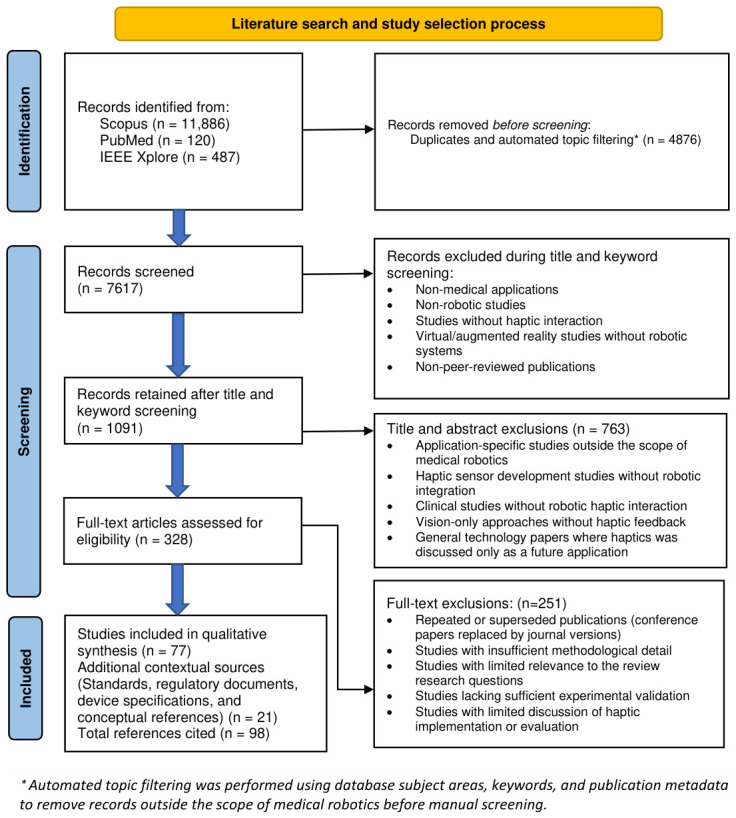
Literature search and study selection workflow. Searches conducted in Scopus, PubMed, and IEEE Xplore yielded 12,479 records. Following deduplication and automated topic filtering, 7617 records were screened. After title, abstract, and full-text assessment according to predefined inclusion and exclusion criteria, 77 studies were retained for qualitative synthesis. 21 additional contextual sources, including standards, regulatory documents, and device specifications, were incorporated to support the discussion of clinical translation and system implementation.

The PRISMA 2020 flow diagram summarizing the identification, screening, eligibility assessment, and inclusion of studies is presented in Figure 1. PRISMA Checklist can be found in the Supplementary Material (Table S1). The combined database searches yielded 12,493 records. Exported records were consolidated and subjected to duplicate removal and automated topic filtering using database subject classifications, keywords, and publication metadata. This preliminary filtering stage removed records outside the scope of medical robotics and reduced the dataset for manual screening.

The remaining records underwent title, keyword, and abstract screening using predefined inclusion and exclusion criteria. Inclusion criteria comprised peer-reviewed journal articles, conference papers, reviews, and experimental studies addressing haptic sensing, haptic rendering, force feedback, tactile interaction, teleoperation, rehabilitation robotics, prosthetics, simulation, or human–robot interaction within a medical context. Exclusion criteria included publications unrelated to medical robotics, studies without a robotics component, papers lacking a haptic interaction element, virtual or augmented reality studies without robotic systems, non-peer-reviewed publications, and publications outside the English language.

Subsequent title, abstract, and full-text assessment further excluded studies that focused primarily on application-specific domains outside the scope of medical robotics, haptic sensor development without robotic integration, clinical procedures without robotic haptic interaction, vision-only approaches, or technologies in which haptics was discussed only as a potential future application. Additional exclusions were applied to superseded conference publications when substantially expanded journal versions describing the same work were available, as well as studies with insufficient methodological detail or limited relevance to the review research questions.

Following the screening and eligibility assessment process, 77 studies were retained as the primary evidence base for the qualitative synthesis presented in this review. To complement these studies, 21 additional contextual sources—including standards, regulatory documents, device specifications, and conceptual references—were incorporated to support discussions related to system implementation, safety, and clinical translation. Consequently, the final manuscript cites 98 references.

Because the included studies differed substantially in design, application domain, evaluation metrics, and outcome measures, a qualitative thematic synthesis was performed instead of a quantitative meta-analysis.

### 1.5. Review Structure

The remainder of this review is organized as follows. Section 2, Haptic Perception and Device Technologies, introduces the physiological and engineering foundations of haptic interaction. It comprises Section 2.1, The Physiology of Touch; Section 2.2, Sensorimotor Integration; Section 2.3, From Perception to Device Design; Section 2.4, Device Classes for Haptic Interaction; and Section 2.5, Evaluation and Performance Metrics.

Section 3, Principles of Haptic Rendering, presents the theoretical foundations underlying haptic interaction, including Section 3.1, The Haptic Interaction Loop; Section 3.2, Transparency: Fidelity of Interaction; Section 3.3, Stability: Safety of Interaction; Section 3.4, Balancing Transparency and Stability; Section 3.5, Control Architectures; Section 3.6, Perceptual and System Constraints; and Section 3.7, Design Best Practices.

Section 4, Haptic Feedback in Medical Robotics, reviews the implementation of haptic feedback in medical robotic systems through Section 4.1, Role and Benefits of Haptic Feedback; Section 4.2, Feedback Modalities and Cue Encoding; Section 4.3, Distributed and Wearable Feedback Systems; Section 4.4, Control Strategies for Medical Haptics; Section 4.5, Representative Systems and Case Studies; and Section 4.6, Integration Challenges.

Section 5, Applications in Medicine and Healthcare, surveys major application domains, including Section 5.1, Surgical and Teleoperated Robotics; Section 5.2, Rehabilitation and Diagnostic Systems; and Section 5.3, Training, Simulation, and Prosthetics.

Section 6, Challenges and Research Frontiers, discusses the remaining barriers to clinical translation through Section 6.1, Technical Challenges; Section 6.2, Human-Factor Challenges; and Section 6.3, Safety, Reliability, and Regulatory Translation.

Section 7, Future Directions in Medical Haptics, outlines emerging research opportunities through Section 7.1, Data-Driven Haptics and Sensorless Force Estimation; Section 7.2, Emerging Interface Technologies; and Section 7.3, Outlook and Research Priorities. Finally, Section 8 concludes the review by summarizing the key findings, discussing their clinical implications, and presenting recommendations for future research and translation.

## 2. Haptic Perception and Device Technologies

Touch is a distributed sensory modality that combines multiple afferent channels to encode pressure, vibration, temperature, stretch, and force. In humans, haptic perception arises from the integration of tactile and kinesthetic information, both of which continuously interact with motor control pathways. Understanding this dual pathway is crucial for designing effective medical haptic interfaces.

### 2.1. The Physiology of Touch

#### 2.1.1. Cutaneous Sensation

**Figure 2:**
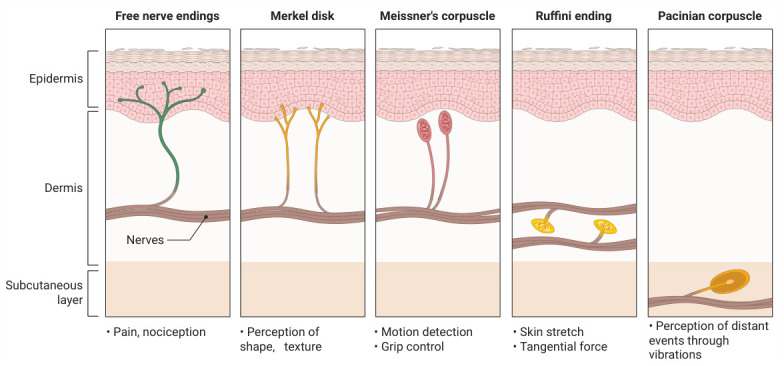
Representative cutaneous mechanoreceptors involved in human haptic perception. Merkel cells, Meissner corpuscles, Ruffini endings, and Pacinian corpuscles exhibit distinct sensitivities to pressure, vibration, skin stretch, and texture, forming the physiological basis for tactile feedback systems discussed throughout this review [5,6].

The cutaneous subsystem consists of mechanoreceptors embedded within the skin (Figure 2), each tuned to specific spatial and temporal features (Table 2). These receptors relay information via fast-conducting A-β afferents to the somatosensory cortex, where the brain reconstructs surface geometry and texture. When a surgeon manipulates tissue, minute variations in vibration and compliance are encoded by these receptors, providing immediate cues about stiffness and thus pathology.

#### 2.1.2. Kinesthetic Sensation

The kinesthetic subsystem is mediated by muscle spindles, Golgi tendon organs, and joint mechanoreceptors. It conveys information about limb position, motion, and effort the internal sense of body configuration known as proprioception. Kinesthesia is essential for applying appropriate force when gripping or suturing. In robotic teleoperation, kinesthetic feedback is reproduced by applying controlled resistance or torque through actuators, allowing users to sense remote forces as if directly connected to the environment.

### 2.2. Sensorimotor Integration

Touch and movement are inseparable. The nervous system forms a closed-loop feedback in which sensory feedback refines motor commands. When an instrument slips, for example, tactile sensors detect the event and trigger reflexive grip adjustments. This tight coupling between perception and action inspired the “active touch” paradigm [6], which posits that humans explore and learn object properties through purposeful movement.

Haptic device designers emulate this principle. They build systems that measure user motion (input) and render reaction forces (output) in real time, effectively recreating the biological feedback loop. The degree of synchrony between action and response largely determines the realism of the interaction.

### 2.3. From Perception to Device Design

#### 2.3.1. Mapping Modalities to Devices

Each sensory mode corresponds to a class of hardware that stimulates the same perceptual channel:

- **Cutaneous → tactile devices**: simulate skin deformation, vibration, or temperature through miniature actuators.
- **Kinesthetic → force-feedback devices**: generate resistive forces or torques proportional to motion and environment dynamics.
- **Hybrid systems**: combine both channels to deliver simultaneous cues (e.g., a glove providing fingertip vibration while an arm exoskeleton produces global resistance).

This mapping ensures that the artificial feedback aligns with natural neural coding, reducing cognitive dissonance and improving user embodiment [4].

#### 2.3.2. Design Parameters

The fidelity of a haptic display depends on spatial resolution, temporal resolution, dynamic range, and latency. For cutaneous displays, practical taxel spacing is commonly on the order of a few millimeters for fingertip arrays, reflecting both human tactile spatial limits and engineering constraints [7]. Temporal fidelity is modality dependent: vibrotactile cues target tens-to-hundreds of Hz sensations, while kinesthetic haptic rendering typically relies on high servo update rates (often 500–1000 Hz) to achieve stable, crisp force interaction [8]. Dynamic range and safety limits must be chosen for the intended clinical task because tool–tissue forces vary widely across procedures, and latency should be minimized since delay reduces responsiveness and can constrain stable feedback gains, especially in networked teleoperation.

### 2.4. Device Classes for Haptic Interaction

**Figure 3:**
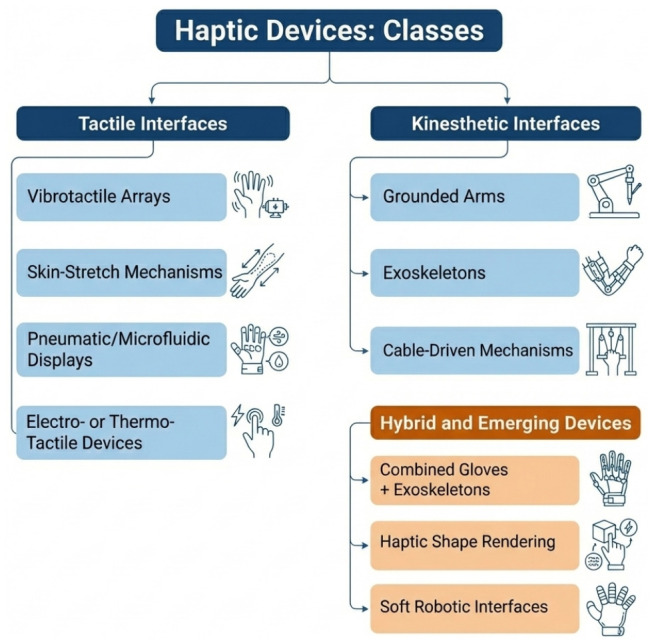
Classification of haptic device technologies according to the primary sensory modality stimulated. Cutaneous interfaces target skin mechanoreceptors through localized tactile stimulation, kinesthetic interfaces render forces and torques through interaction with muscles and joints, and hybrid systems combine both channels to increase information richness and perceptual realism. The classification reflects the hardware-oriented taxonomy discussed in Section 2.4 [5,6,9]. Representative medical applications of these interface categories are discussed in Section 5.

Haptic interfaces can be classified by the primary human sensory channel they stimulate (Figure 3): cutaneous (tactile) devices target skin mechanoreceptors, kinesthetic devices apply forces/torques to muscles and joints, and hybrid devices combine both [5,6,9]. In this section, we focus on the hardware perspective (what devices physically do and the performance limits they impose). Section 4.2 revisits these channels from an information design perspective (what feedback is communicated and how it is encoded in medical tasks).

#### 2.4.1. Cutaneous (Tactile) Interfaces

Cutaneous displays deliver localized stimuli to the skin to reproduce sensations such as vibration, indentation, shear, or temperature, matching the operating characteristics of skin mechanoreceptors and supporting event- and surface-related cueing [5,6,9]. These devices are attractive in medical robotics because they can be compact and wearable, and can convey salient cues (e.g., contact onset, threshold exceedance) without requiring large net forces at the arm. Common implementations include vibrotactile actuation (ERM/LRA or piezoelectric), lateral skin stretch mechanisms for tangential/shear cues [10], normal-indentation modules (electromechanical or pneumatic) [11], and thermal elements (typically thermoelectric) [12]. In practice, tactile devices are limited in net force output and contact area; they are therefore best viewed as cue displays rather than substitutes for full force reflection in tasks where gross forces or torques must be rendered [13].

#### 2.4.2. Kinesthetic Interfaces

Kinesthetic interfaces generate controlled forces and, in some designs, torques at the hand, wrist, or arm, thereby stimulating proprioceptive pathways and enabling mechanical interaction with virtual or remote environments [4,14]. In medical robotics, these devices are commonly used as master interfaces for teleoperation, surgical simulation, and rehabilitation systems requiring force rendering and impedance interaction [4,13].

From a hardware perspective, three families dominate. Grounded desktop manipulanda provide high bandwidth and stable force rendering but restrict the user to a fixed workspace [4,15]. Upper-limb exoskeletons distribute actuation across multiple joints and are frequently used in rehabilitation and assistive applications, where backdrivability and compliant interaction are important for safety [16,17]. Cable-driven systems offer larger workspaces with low moving inertia, making them attractive for teleoperation scenarios that require wide reach and reduced reflected mass [18]. Depending on the application, resistance forces may be generated using either active or passive mechanisms. Active systems commonly employ electric motors (e.g., direct-drive or linear motors) to render programmable forces and virtual environments, whereas passive devices typically rely on controllable brakes, friction mechanisms, or magnetic particle brakes to resist user motion without injecting energy into the interaction. Passive approaches offer inherent safety and robustness, while active actuation provides greater flexibility for realistic force rendering and teleoperation.

Compared with cutaneous devices, kinesthetic interfaces generally provide greater force authority and impedance rendering capability, although this comes at the cost of increased size, weight, and integration complexity [4,14].

#### 2.4.3. Hybrid and Emerging Interfaces

Hybrid interfaces combine cutaneous and kinesthetic hardware within a single system to exploit the complementary capabilities of both modalities [9]. Typical implementations pair force-generating devices such as grounded manipulanda or exoskeletons with fingertip tactors, skin stretch cues, or wearable tactile displays. Hybrid designs are particularly attractive in medical robotics because they can increase perceptual richness without relying exclusively on large kinesthetic forces.

Emerging directions include soft wearable actuators for rehabilitation and assistive applications [19], as well as contactless ultrasound-based mid-air haptics that provide tactile interaction without physical contact [20]. In many medical scenarios, hybrid systems offer a practical compromise between realism, safety, comfort, and system complexity, particularly when distal sensing or high-authority force reflection is difficult to implement [13,21].

Beyond the sensory modality classification presented above, haptic interfaces can also be categorized according to their interaction mechanism as isometric or isotonic devices. Isometric interfaces measure user-applied forces while allowing little or no displacement, making them suitable for applications requiring precise force control, such as rehabilitation assessment and grip-force training. In contrast, isotonic interfaces permit continuous user motion while rendering forces or torques, making them the dominant architecture for teleoperation, surgical simulation, and robot-assisted manipulation. Although both approaches provide haptic interaction, isotonic devices generally offer higher realism for movement-based tasks, whereas isometric interfaces are often simpler, more compact, and advantageous when force measurement is the primary objective.

### 2.5. Evaluation and Performance Metrics

Evaluating haptic interfaces requires both system-level metrics (what the device and controller can physically and stably render) and human/task-level outcomes (what users can perceive and how well they perform). On the system side, a foundational capability descriptor is the device’s renderable impedance range. Colgate and Brown’s Z-width concept captures this range by characterizing how well a device can render very low impedances (transparent free-space motion) and very high impedances (stiff contact) while remaining stable [22]. In practice, Z-width is strongly influenced by mechanical design (inertia, friction, damping), actuator authority, and discrete-time effects (sampling and delay).

Complementary system metrics quantify how accurately commanded interactions are delivered. Force tracking error compares the commanded versus measured output force during representative motions and contacts and is frequently used to benchmark controller performance and bandwidth. Because medical tasks are ultimately limited by human perception, psychophysical measures also matter: just noticeable differences (JNDs) for force and stiffness provide practical bounds on the resolution that is likely to be perceptually meaningful (e.g., typical Weber fractions on the order of ΔF/F and Δk/k) [23,24]. Finally, because realism and clinical utility are task-dependent, user studies remain essential. Typical outcomes include completion time, accuracy/error rates, peak and mean applied forces (safety-related), learning curves in training scenarios, and subjective ratings of realism, workload, and comfort.

In medical contexts, performance targets are typically framed as a balance between fidelity and safety: devices should provide sufficiently accurate and consistent cues to support the intended clinical or training task, while guaranteeing stable, fail-safe operation under user variability and environmental uncertainty.

## 3. Principles of Haptic Rendering

Haptic rendering is the computational and control process that transforms sensory data and mathematical models into tactile or force sensations a user can feel. In a medical context, rendering enables a clinician to “touch” virtual or remote tissues through an interface by calculating and reproducing realistic interaction forces in real time. The performance of haptic rendering systems is governed by transparency and stability, as discussed below.

### 3.1. The Haptic Interaction Loop

Building on the biological sensorimotor loop described in Section 2, haptic rendering systems implement an engineering analog that couples the user, device, and environment. The typical sequence is:

1. Human motion: the user moves the handle or stylus.
2. Sensing: encoders or force sensors measure position x(t), velocity v(t), and applied force F(t).
3. Computation: a controller or simulation computes the environment’s response (virtual stiffness, damping, collision).
4. Actuation: motors or actuators render the calculated reaction force $F_{r}$ back to the user.

This cycle repeats at high frequency; update rate is critical. Whereas vision operates comfortably at ≈60 Hz, realistic force feedback demands ≥ 1 kHz update to avoid perceivable lag or oscillation [4]. In teleoperated leader–follower surgical systems, this loop extends over a communication channel, introducing latency (Δt ≈ 5–100 ms) and possible packet loss, which must be compensated through control design.

A simple linear approximation of the interaction force is given by:

(1) $$F_{r}\left(t\right)\;=\;K\left(x_{r}\;-\;x_{u}\right)\;+\;B\left({\dot{x}}_{r}-\;{\dot{x}}_{u}\right)$$

where K and B are virtual stiffness and damping, $x_{r}$ is the reference (environment position), and $x_{u}$ the user input. This virtual spring–damper formulation is the foundation of impedance-type rendering. In many practical haptic rendering applications, particularly when rendering stationary virtual environments, the reference environment does not move and therefore $\dot{x_{r}}=0$. Under this common assumption, Equation (1) simplifies to

$$F_{r}=K\left(x_{r}-x_{u}\right)-B{\dot{x}}_{u},$$

where the damping term depends solely on the user velocity. The more general formulation given in Equation (1) is retained because it also applies to moving reference environments and bilateral teleoperation scenarios, where the reference velocity ${\dot{x}}_{r}$ may be non-zero.

### 3.2. Transparency: Fidelity of Interaction

Transparency quantifies how accurately the rendered impedance $Z_{d}\left(s\right)$ matches the true environment impedance $Z_{e}\left(s\right)$. Impedance is defined as the ratio between interaction force and velocity in the Laplace domain,

(2) $$Z\left(s\right)=\frac{F(s)}{V(s)}\;\;$$

where F(s) and V(s) denote the Laplace transforms of the interaction force and velocity, respectively, and *s* is the Laplace variable. Perfect transparency implies that an operator cannot distinguish between direct physical contact and interaction mediated through a haptic interface ($Z_{d}\left(s\right)\;=\;Z_{e}\left(s\right)$). In practice, this ideal is unattainable due to physical and computational constraints, including sensor resolution limits, actuator friction, signal quantization, and communication or control delays.

Consequently, perceptual fidelity is commonly evaluated using objective measures such as force tracking error and rendered-to-target stiffness ratios, as well as task-based performance metrics (e.g., palpation accuracy). Experimental studies in soft-tissue telemanipulation have shown that reliable stiffness perception can be achieved even in the presence of significant deviations from ideal transparency, provided that feedback is stable, consistent, and preserves relative stiffness cues [25].

### 3.3. Stability: Safety of Interaction

While transparency targets perceptual realism, stability is the primary safety requirement for any haptic interface that exchanges power with a human user. A widely used framework for stability analysis is passivity theory, which provides a conservative but practical sufficient condition for stable interaction with an unknown (and potentially active) human operator and environment. Informally, a passive system cannot generate net energy: the cumulative energy delivered to the user must not exceed the energy initially stored in the system. In a one-port haptic interaction, this can be written as an inequality on the exchanged energy

(3) $$E\left(t\right)\;=\;\int_{0}^{t}F\left(\tau \right)\;\dot{x}\left(\tau \right)\;d\tau \;\geq \;-E_{0},$$

where F(τ) is the interaction force, $\dot{x}\left(\tau \right)$ is the interface velocity, and $E_{0}\geq 0$ is the initial stored energy. (If the system starts with zero stored energy, $E_{0}=0$ and E(t)≥0). This energy-based view is particularly useful in haptics because digital implementation details (including sampling, quantization, computation delay, and actuator/sensor dynamics) can inject non-physical (“virtual”) energy that manifests as oscillations, buzzing, or instability during contact rendering [22,26,27].

In networked haptic teleoperation, the stability problem is amplified by time delay and jitter in the communication channel. Even moderate delays (often on the order of tens of milliseconds and above, depending on controller gains and device dynamics) can destabilize high-gain bilateral force reflection unless the architecture explicitly accounts for energy flow [18,27]. For this reason, many practical systems intentionally trade some transparency for robust stability margins via passivity-preserving or passivity-enforcing designs.

Rather than listing techniques, it is often clearer to view stabilization methods as variations on a single principle: prevent net energy generation at the human port. Time-domain passivity observers/controllers (PO/PC) monitor the exchanged energy and inject dissipation (typically adaptive damping) only when necessary to maintain passivity [27]. Communication-channel passivation approaches (e.g., scattering/wave-variable transformations) reparameterize the exchanged signals so that the channel remains passive despite delay, which is why they are a cornerstone of bilateral teleoperation under delay [18]. Energy-tank methods extend the same idea by explicitly budgeting energy: the controller may render high impedance only when sufficient “stored” energy is available, thereby preserving passivity while allowing more aggressive interactions when safe [28]. In real medical systems, detailed control implementations are often proprietary, but commercial platforms typically adopt conservative gain limits, safety monitors, and fault-handling modes to reduce the risk of unstable force reflection under uncertainty (delay variation, sensor dropout, and user variability), which is consistent with general safety-driven design practice in medical robotics.

### 3.4. Balancing Transparency and Stability

A central design tension in haptics is that transparency and stability are inherently coupled: increasing the rendered stiffness (e.g., a “harder” virtual wall) improves realism but reduces stability margins in discrete-time, actuator-limited systems. Classic analyses of the Z-width of a haptic display show that the maximum stably renderable impedance is finite and is strongly shaped by sampling rate, delay, and the device’s physical damping/inertia [22,26]. As a result, practical haptic systems are often engineered so that both hardware and control reduce the likelihood of energy injection at the human port.

From the hardware side, low apparent inertia and friction (i.e., low intrinsic mechanical impedance) increase achievable transparency in free space and permit higher stable contact stiffness under the same sampling/actuation constraints. From the control side, higher servo rates (often ≳1 kHz for kinesthetic rendering) and careful discrete-time design improve stability margins and reduce artifacts during contact [22,27]. When additional uncertainty is present (e.g., variable delay in teleoperation, or changing interaction dynamics), adaptive strategies may tune virtual coupling gains or damping online to avoid instability while retaining useful feedback; learning-based methods are increasingly explored in this context, but they must still respect passivity/safety constraints in medical use [29]. Finally, perceptually optimized damping is often used in practice: it adds only as much dissipation as needed to suppress oscillations, ideally remaining below the threshold where the interaction feels noticeably sluggish.

### 3.5. Control Architectures

Two canonical control paradigms dominate haptic rendering and largely reflect the intrinsic mechanical impedance of the device and the sensing available: impedance control and admittance control (see Table 3). In impedance control (classically introduced in robotics by Hogan), the controller maps measured motion to a commanded interaction force,

(4) $$F\;=\;Kx+B\dot{x}+M\ddot{x},$$

and is therefore best suited to low-inertia, backdrivable devices that can accurately render forces in response to user motion. In admittance control, the controller measures the interaction force and computes a motion response through a virtual dynamical model, which is often preferable when the device/robot has higher inertia, friction, or limited backdrivability (because it is easier to regulate motion than to achieve high-fidelity force output under those conditions) [30].

Hybrid designs blend or switch between these paradigms based on interaction state (e.g., free-space versus contact) to exploit their complementary strengths: admittance-like behavior can improve robustness in free motion, while impedance-like behavior can provide more direct and transparent contact rendering when conditions allow [30]. This trade-off is particularly relevant in medical robotics, where both stable free-space maneuvering and safe, informative contact interaction are required.

### 3.6. Perceptual and System Constraints

Human haptic perception imposes practical limits on the useful resolution and timing of rendered feedback. In many medical-robotic tasks, improving force/impedance rendering beyond human discrimination thresholds yields diminishing perceptual benefit, while often increasing control complexity and risking reduced stability margins. For this reason, it is common to use representative psychophysical thresholds as design targets (Table 4), while recognizing that the exact values depend strongly on factors such as exploration strategy (active vs. passive), contact conditions, direction of loading, reference force/stiffness level, and user training.

From the engineering side, two system-level limits largely determine how “real” the rendered interaction can feel:

1. Maximum stable renderable stiffness, $K_{\max}$ (N/m): This is the highest virtual contact stiffness that can be rendered without oscillation or instability. In practice, $K_{\max}$ is limited by the haptic servo rate $f_{s}$ (Hz), total loop delay, actuator force authority, and the device’s intrinsic damping, inertia, friction, and quantization/noise.
2. Minimum renderable impedance, $Z_{\min}$: Even with “zero” commanded force, the user still feels the device’s intrinsic impedance due to inertia, viscous/Coulomb friction, transmission backlash, and sensor/actuator imperfections. If this baseline impedance is too high, users perceive a “sticky” or “heavy” free-space motion.

As defined in Section 2.5, Z-width summarizes the stable renderable impedance range; wider Z-width generally enables more realistic free-space motion and firmer contact rendering. A wider Z-width generally indicates a more capable interface for medical robotics, because it can better support both near-zero impedance behavior for precise free-space manipulation and high-impedance contact rendering for palpation, constraint interaction, and “hard” tool–tissue events [26].

### 3.7. Design Best Practices

The following best practices represent direct design responses to the perceptual and system constraints outlined above:

1. Prioritize safety and passivity: stability always overrides realism.
2. Optimize mechanical design: lightweight, low-friction mechanisms yield better transparency.
3. Use high-rate control loops: for both rendering and sensor readout.
4. Employ adaptive damping/gain scheduling: based on user motion or estimated delay.
5. Synchronize multimodal cues: align haptic, visual, and auditory feedback (desynchronization degrades performance).
6. Validate perceptually: user studies remain the final benchmark of fidelity and comfort.

## 4. Haptic Feedback in Medical Robotics

Haptic feedback closes the perceptual loop between a clinician and the robot, enabling bidirectional communication: the operator commands motion and simultaneously perceives the reaction forces from the patient or virtual environment. In medical robotics, this feedback transforms robotic systems from purely visual teleoperators into multisensory extensions of the human hand, enhancing precision, safety, and situational awareness.

When implemented correctly, haptics enables a surgeon to distinguish tissue stiffness, identify boundaries, feel instrument vibration, and control grip force intuitively, all of which are lost in conventional teleoperative minimally invasive or robot-assisted surgery (RAS).

### 4.1. Role and Benefits of Haptic Feedback

Haptic feedback provides several benefits, including force perception, event detection, skill augmentation through guidance, safety enhancement, and reduction of visual and cognitive workload. Empirical studies and meta-analyses consistently show that adding haptic feedback in robot-assisted surgical tasks reduces applied forces and often improves completion time and accuracy, although the magnitude depends on task type and user expertise. For example, a recent meta-analysis reported large overall effects of haptic feedback on applied force and completion time and a very large effect on accuracy [1,13,32].

### 4.2. Feedback Modalities and Cue Encoding

As discussed in Section 2.4, medical haptic systems employ cutaneous, kinesthetic, and hybrid hardware interfaces. From an application perspective, however, the more important distinction concerns the information conveyed to the user rather than the device itself. In medical robotics, haptic feedback is typically designed to communicate specific task-relevant information while remaining interpretable, stable, and compatible with clinical workflow [13,14]. The principal characteristics of the three major feedback modalities are summarized in Table 5. Rather than representing competing technologies, these modalities often provide complementary channels for conveying clinically relevant information.

As shown in Table 5, each modality occupies a distinct role in medical robotics. Kinesthetic feedback is most suitable for conveying gross interaction mechanics and guidance forces, cutaneous feedback excels at communicating localized events and warnings, while hybrid approaches seek to combine the advantages of both channels. This distinction motivates the information-centric perspective adopted throughout this review, in which the choice of feedback modality is guided by the information that must be conveyed rather than by hardware considerations alone.

#### 4.2.1. Cutaneous Cueing: Event and Surface Information

Cutaneous feedback is primarily used to communicate localized, high-salience information that does not require large force output. Typical examples include contact onset and offset, puncture events, tool–bone contact, slip direction, and threshold exceedance warnings [5,9,10]. Because these cues are intended to support rapid perception under varying glove and skin conditions, they are often encoded as discrete signals such as pulses, bursts, or directionally coded patterns rather than continuous force representations [9]. Careful calibration remains important because excessive cue intensity can increase distraction, workload, and fatigue [33].

#### 4.2.2. Kinesthetic Feedback: Force and Guidance Information

Kinesthetic feedback is used when the objective is to communicate gross interaction mechanics, including force magnitude, compliance, stiffness changes, or motion constraints [13,14]. In bilateral teleoperation, this information is commonly delivered through force reflection or virtual coupling strategies that preserve stable contact rendering under sensing and communication limitations [18,27,34]. In shared-control applications, kinesthetic feedback can also encode virtual fixtures and guidance fields, helping users follow safe trajectories or avoid sensitive regions without requiring direct reproduction of every contact force [35,36]. In all cases, stability and predictability generally take precedence over maximum achievable stiffness [18,27].

#### 4.2.3. Multimodal Cue Allocation

Combining cutaneous and kinesthetic feedback allows information to be distributed according to modality strengths. A common strategy is to convey low-frequency force and stiffness information through the kinesthetic channel while reserving cutaneous feedback for high-frequency events such as contact transitions, slip, alarms, and safety warnings [10]. Multimodal designs can also provide redundant communication of critical events, improving detection under visual and cognitive load [33]. Examples include hybrid systems that combine kinesthetic master devices with fingertip tactors for contact cueing, tactile augmentation of haptic shape rendering [37], and emerging soft wearable interfaces that improve comfort during prolonged interaction [9]. The central design objective is not necessarily maximum realism, but efficient distribution of information across modalities while maintaining stability, usability, and clinical relevance.

### 4.3. Distributed and Wearable Feedback Systems

Traditional grounded haptic interfaces (e.g., desktop kinesthetic devices) can deliver high force bandwidth and stable impedance rendering, but their fixed bases restrict workspace and complicate integration into clinical workflows (e.g., space constraints, sterility boundaries, and multi-user setups). These limitations have motivated distributed and wearable haptic systems, which place actuators closer to the user’s body and deliver information through compact, lightweight mechanisms. In the context of medical robotics, the value proposition is often practical rather than purely perceptual: wearable systems can be donned quickly, used in larger workspaces, and—depending on design—kept outside the sterile field while still transmitting task-relevant cues to the operator.

A prominent category is hand-worn or glove-based feedback. Such devices typically combine fingertip vibrotactile cues (contact onset, threshold exceedance, slip events) with mechanisms that provide limited kinesthetic effects such as grasp resistance or braking. For example, commercial glove platforms report force-feedback capability on the order of tens of newtons for grasp assistance/resistance along with vibrotactile feedback for event cueing (e.g., the SenseGlove Nova 2 product specifications) [38,39]. In medical teleoperation and training, glove-based systems are most effective when the haptic message is designed around interpretable cues (contact, slip direction, “do-not-exceed” force boundaries) rather than full force reflection, which remains difficult to realize safely and transparently in lightweight wearables. Recent developments in wearable rehabilitation technologies further demonstrate the potential of glove-based haptic systems. For example, soft robotic gloves with integrated sensing and force feedback have been proposed for rehabilitation and virtual-reality-based training, providing both assistive interaction and objective performance assessment [40]. Although most current studies remain limited to pilot evaluations and controlled user experiments, these systems illustrate a promising pathway toward lightweight and home-compatible haptic rehabilitation.

A second direction is body-worn haptics (e.g., sleeves, armbands, vests), which deliberately redistributes haptic information away from the hand. This approach is particularly relevant for immersive simulation, telepresence, and guidance scenarios, where the objective is to convey directional cues, warnings, or state transitions without overloading the operator’s fingertips or constraining fine motor control. In practice, body-worn systems commonly encode information using vibrotactile patterns distributed across larger skin areas, trading fine spatial resolution for robustness, comfort, and interpretability. Reviews of wearable and cutaneous haptic systems emphasize that such designs are highly effective for communication and guidance, even when they are not intended to reproduce high-fidelity contact mechanics [9].

A third emerging modality is mid-air (contactless) haptics, most commonly implemented using phased ultrasonic arrays. These systems focus ultrasound to create perceivable tactile sensations without physical contact, which is attractive for hygiene-sensitive interfaces and touchless interaction concepts. Classic demonstrations show that ultrasonic phased arrays can render spatially and temporally modulated tactile stimuli in mid-air, enabling “virtual buttons,” contours, and event cues (e.g., Carter et al.) [20]. However, mid-air haptics should generally be framed as a tactile cueing channel: the achievable effective forces at the skin are limited compared with grounded kinesthetic devices, and the most compelling use cases are typically notifications, contact/event signaling, or simple shape/texture illusions rather than high-magnitude force feedback. While airborne ultrasonic haptics has demonstrated reliable tactile cue generation and encouraging results in human-subject studies, evidence of large-scale clinical deployment remains limited. Current applications are primarily focused on guidance, warning cues, and sterile human–machine interaction rather than direct therapeutic intervention or high-authority force rendering [20].

Overall, wearable and distributed haptics expand deployability and reduce setup constraints, but they usually trade away maximum renderable impedance and force bandwidth. For medical robotics, this trade-off is often acceptable—especially when the interface is designed around task-relevant cues (events, thresholds, slip direction, guidance) and is validated for usability, comfort, and safety in realistic clinical workflows. Early rehabilitation studies using wearable force-feedback systems and soft robotic devices further suggest that clinically meaningful haptic interaction can be achieved without the large, grounded hardware traditionally associated with force feedback [40,41].

### 4.4. Control Strategies for Medical Haptics

The control architecture determines how signals acquired from force, position, velocity, or estimated interaction sensors are processed into haptic commands rendered by the user interface. In medical robotics, these algorithms transform measured or estimated tool–tissue interactions into stable, perceptually meaningful force or tactile feedback while accounting for sensing uncertainty, communication delay, and safety constraints. Rather than enumerating strategies as disconnected items, it is helpful to view the major approaches as points on a spectrum: from direct physical force reflection (highest transparency potential, highest sensitivity to delay/noise) to task-aware cueing and guidance (lower physical fidelity, often higher robustness and easier deployment).

#### 4.4.1. Direct Force Reflection (Bilateral Teleoperation)

In bilateral control, the follower-side interaction force $F_{f}$ (measured or estimated) is rendered at the master interface as a command $F_{m}$, often in a scaled form,

(5) $$F_{m}=k\;F_{f},$$

where *k* is a scaling factor chosen for perception, safety, or workspace normalization. When the sensing is reliable and delays are small, bilateral reflection can provide highly intuitive interaction. In practice, however, force reflection is sensitive to sensor noise, quantization, and time delay, and can become unstable if the overall loop injects energy. As a result, most medical-robotic implementations apply filtering and conservative gain design, and frequently incorporate passivity-enforcing methods such as time-domain passivity control (PO/PC) to maintain stable contact across operating conditions [27,42]. For networked teleoperation, wave/scattering transformations remain a foundational tool for preserving passivity over delayed channels and therefore for stabilizing force feedback under non-negligible latency [18].

#### 4.4.2. Shared Control, Virtual Fixtures, and Guidance Fields

A widely used alternative (or complement) to force reflection is to render guidance rather than raw interaction forces. Virtual fixtures constrain motion within safe regions or along desired paths, producing forces that “nudge” the operator toward a target while still allowing voluntary control. In its simplest blending form, the commanded haptic cue can be written as

(6) $$F_{c}=\alpha \;F_{h}+\left(1-\alpha \right)\;F_{a},$$

where $F_{h}$ is the user-command contribution, $F_{a}$ is an autonomous assistive/constraint force (e.g., from a fixture or planner), and α∈[0,1] controls the arbitration level. In surgical and interventional contexts, virtual fixtures are attractive because they can reduce error and enforce safety constraints even when distal force sensing is limited, and because the haptic message is directly tied to task structure (e.g., forbidden regions, target corridors) [35].

#### 4.4.3. Model-Based and Data-Driven Rendering (Sensorless Haptics)

Because integrating sterilizable multi-axis distal force sensors remains challenging for many instruments, a growing body of work relies on force estimation and model-mediated haptics. Here, the feedback is computed from analytical tissue models, robot dynamics (motor currents plus calibrated friction/inertia), or learned mappings from data (e.g., vision-based deformation to force). The goal is not necessarily perfect physical reconstruction, but rather consistent and informative cues that preserve stability and help the operator discriminate clinically relevant interaction states. In medical robotics, model-mediated strategies are often positioned as a practical route to haptic augmentation when direct sensing is infeasible, and recent learning-based approaches are increasingly used to improve estimation robustness and context awareness [43,44].

#### 4.4.4. Latency Compensation and Practical Robustness

Across all architectures, latency compensation is central when teleoperation or distributed computation is involved. Predictive elements, rate limiting, and passivity-based methods are frequently combined to maintain stable interaction and prevent oscillations when delay varies. Importantly, the control objective in safety-critical medical tasks is often robustly stable, interpretable feedback rather than maximal transparency, particularly when sensing uncertainty or communication delay is significant [18,45].

### 4.5. Representative Systems and Case Studies

**Figure 4:**
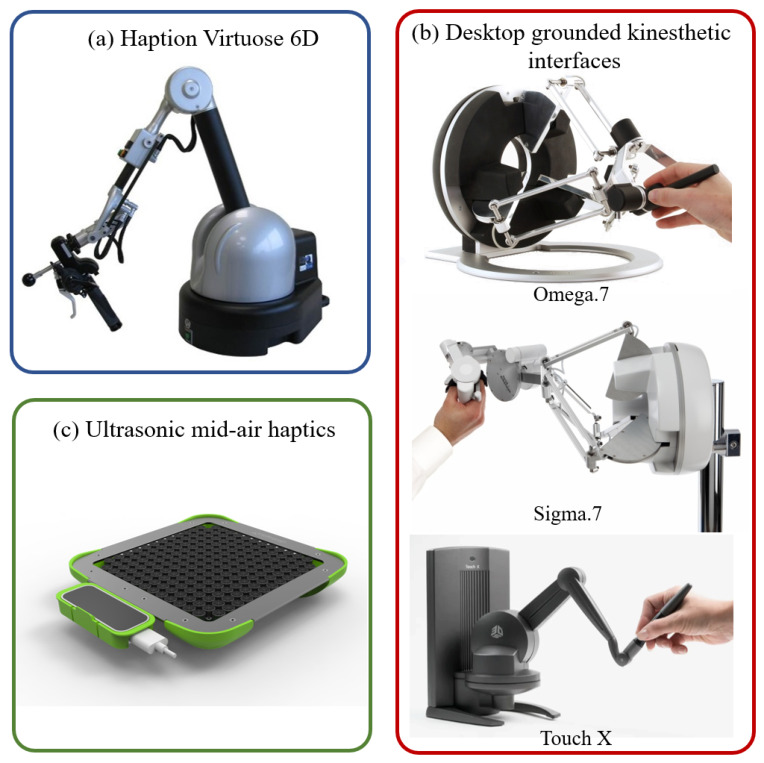
Representative haptic systems used in medical robotics and surgical simulation. (**a**) Haption Virtuose 6D, a grounded 6-DOF kinesthetic interface capable of rendering forces and torques for teleoperation and simulation; (**b**) representative desktop kinesthetic interfaces including Omega.7, Sigma.7, and Touch X, commonly used as master devices in surgical simulation and robotic teleoperation; (**c**) ultrasonic mid-air haptic technology for contactless tactile feedback. These examples illustrate the diversity of current haptic platforms, ranging from high-force kinesthetic systems to lightweight tactile displays [4,15,20].

Several representative commercial and research systems illustrate how haptic sensing, force rendering, and control strategies are translated into practical medical robotic platforms (Figure 4). Rather than providing an exhaustive catalog of devices, the following examples highlight different engineering approaches to haptic implementation, including grounded kinesthetic interfaces, research teleoperation platforms, and clinically deployed surgical robots. These case studies illustrate the practical trade-offs between sensing, control, force rendering, and clinical integration.

1. Haption Virtuose 6D: The Haption Virtuose 6D RV is a grounded 6-DOF kinesthetic interface designed to render both forces and torques with a comparatively large workspace. According to the manufacturer datasheet, the device provides (at workspace center) 35 N peak/10 N continuous force, and 3.1 N·m peak/1 N·m continuous torque, with a 1 kHz update rate. The same datasheet reports a maximum translational stiffness of 2000 N/m (2 N/mm) in the standard configuration, and higher stiffness for high-force configurations [46].
2. Desktop Grounded Kinesthetic Interfaces (Omega.7, Sigma.7, Touch/Touch X): Desktop grounded kinesthetic interfaces constitute one of the most widely used classes of haptic hardware in medical robotics, serving as master devices for teleoperation and interaction interfaces for surgical simulation. Most systems employ serial or parallel linkages equipped with high-resolution position encoders and electric actuators that render translational forces and, in some cases, torques or grasp feedback directly to the surgeon’s hand. Their mechanical design prioritizes low inertia, backdrivability, and high control bandwidth (typically 1–4 kHz) to maximize transparency while maintaining stable interaction. Consequently, these devices can render relatively stiff and stable contact interactions, making them well suited for experimental teleoperation platforms and surgical simulators. Commercial systems such as the Omega.7, Sigma.7, Touch X, and Virtuose 6D exemplify different implementations of these design principles, providing a practical compromise between realism, safety, and deployability [4,15,18].
3. Ultrasonic mid-air arrays (UltraHaptics/Ultraleap): Ultrasonic phased arrays (popularized by UltraHaptics, now within Ultraleap) can generate mid-air tactile sensations, enabling contactless haptic cues. This modality is attractive when physical contact should be minimized (e.g., touchless interfaces in hygiene-sensitive settings), but it should be presented as tactile cueing rather than high-force kinesthetic rendering. A foundational demonstration of multi-point mid-air ultrasound haptics is reported in the UltraHaptics work [20].

As shown in Table 6, commercially available systems span a broad spectrum ranging from research-oriented kinesthetic interfaces to clinically deployed surgical robots and wearable haptic devices. Despite substantial progress, the table illustrates that true force-reflecting surgical systems remain relatively uncommon in clinical practice, while wearable and contactless solutions often prioritize usability and deployment flexibility over maximum force-rendering capability.

### 4.6. Integration Challenges

Translating haptic feedback from laboratory prototypes to clinically usable medical robotic systems requires addressing constraints that are largely external to classical haptic rendering and control theory. In particular, the operating-room ecosystem imposes strict requirements on sterility, instrument form factor, and safety/regulatory compliance, while user variability (e.g., glove use and differences in tactile sensitivity) affects how haptic cues should be designed and calibrated. These constraints help explain why many clinically deployed platforms still provide limited or no true force reflection, even when the technical benefits of haptics have been demonstrated in controlled studies [21].

#### 4.6.1. Sterilization and the Sterile Barrier

Unlike general-purpose haptic devices, medical robotic components that approach the patient must be compatible with cleaning and sterilization workflows. In practice, this often forces a physical separation between sensitive electromechanical components (actuators, bearings, wiring harnesses) and the sterile field, using drapes, sterile adapters, or locating actuation outside the sterile zone and transmitting motion through mechanical linkages. Where sensing is required near the distal end-effector, sterilization compatibility becomes a dominant design constraint: steam autoclaving can expose components to high temperature and pressure (commonly cited conditions are on the order of 134 °C and 200 kPa), while repeated exposure can degrade adhesives, polymer encapsulants, cable jackets, and calibration stability. Consequently, a substantial fraction of force-sensing approaches reported for robot-assisted minimally invasive surgery emphasize packaging, sealing, and sterilization survivability as key bottlenecks for clinical translation [47].

#### 4.6.2. Miniaturization and Instrument Integration

For minimally invasive instruments, integrating miniature multi-axis force, torque, position, and displacement sensing remains one of the greatest engineering challenges. Available cross-sectional area is extremely limited (typical tool diameters are only a few millimeters), yet clinically useful haptics often requires accurate multi-axis sensing, robust strain transmission, and reliable signal routing without compromising mechanical strength, stiffness, sterilizability, or surgeon ergonomics. These geometric constraints complicate co-location of sensors and actuators at the distal tip and frequently motivate alternative architectures such as proximal sensing, sensor fusion, or model-based/sensorless force estimation. Recent syntheses of force-sensing techniques for robotic surgery highlight that even when adequate force resolution is achieved on benchtop setups, integration into surgical tools remains challenging due to packaging, robustness, and sterilization constraints, which collectively slow down translation toward routine clinical deployment [47].

#### 4.6.3. Safety Certification and Quality Management

Medical haptic systems must be engineered and documented within a regulatory-compliant development process and must satisfy electrical/mechanical safety expectations for medical electrical equipment. In practice, this commonly means designing against the International Electrotechnical Commission (IEC) 60601 family of safety standards (for basic safety and essential performance of medical electrical equipment) and operating under a quality management system aligned with International Organization for Standardization (ISO) 13485 (medical devices quality management system (QMS) requirements used for regulatory purposes) [48,49]. These requirements influence both hardware (e.g., isolation, leakage current, fail-safe behavior, mechanical hazards) and software (verification, risk management, traceability), and they increase the engineering effort needed to productize haptic functionality beyond research prototypes.

#### 4.6.4. User Variability: Perception, Gloves, and Calibration

Finally, haptic feedback must be robust to user-to-user variability in tactile sensitivity and to the practical reality of glove usage in clinical environments. Surgical gloves and double-gloving practices can measurably reduce tactile sensibility and alter perception of fine cues, which has direct implications for how vibrotactile thresholds, indentation amplitudes, and force-scaling should be tuned for reliable perception without annoyance or fatigue [50,51]. This motivates adaptive calibration strategies (or task-specific cue designs) that emphasize perceptually salient, interpretable cues rather than attempting to maximize raw physical fidelity under all conditions.

Overall, these integration constraints mean that clinically viable medical haptics is often less about achieving the highest theoretical transparency and more about delivering stable, safe, sterilization-compatible, and perceptually meaningful cues within tight packaging and regulatory constraints.

## 5. Applications in Medicine and Healthcare

**Figure 5:**
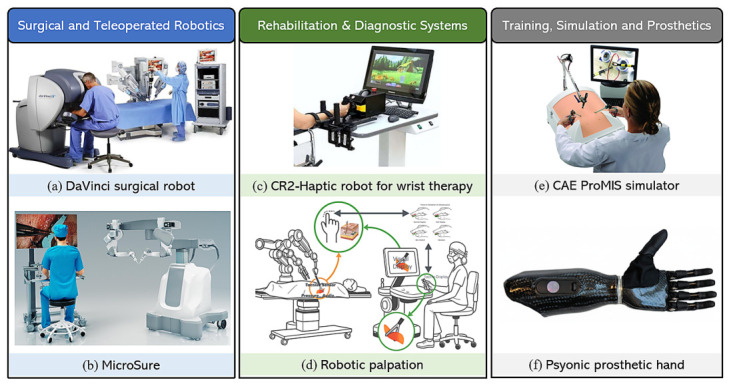
Overview of major application domains of haptics in medical robotics. Haptic feedback supports safe and precise interaction in surgical and teleoperated systems (**a**,**b**), rehabilitation using wearable kinesthetic interfaces and tactile diagnostics through controlled force and tactile cues, (**c**,**d**), and enhances training realism and sensory restoration in simulation platforms and advanced prosthetic devices (**e**,**f**) [17,52,53,54,55].

Haptic technology permeates nearly every branch of medical robotics (Figure 5). It bridges the gap between human perception and robotic control, reintroducing the clinician’s natural sense of touch into teleoperated, assistive, or simulated systems.

### 5.1. Surgical and Teleoperated Robotics

#### 5.1.1. Restoring Force Perception in Surgery

In minimally invasive or robot-assisted surgery (RAS), surgeons manipulate instruments via long shafts or robotic interfaces that sever direct tactile connection to tissue. The lack of haptic cues can lead to overexertion or misjudgment of stiffness. Haptic-enabled surgical robots counteract this by transmitting reaction forces or vibrations from the surgical site to the operator’s hands through a leader–follower setup.

As summarized in Section 4, haptic feedback substantially reduces applied force and improves task efficiency [1]. In neurosurgery and microsurgery, where forces are typically <0.5 N, high-resolution haptics prevents tissue tearing and supports precise dissection.

#### 5.1.2. Example: da Vinci Research Kit (dVRK)

The da Vinci Research Kit (dVRK) is one of the most widely used open research platforms for investigating haptic feedback in robot-assisted surgery. Built from retired da Vinci Surgical System hardware, it provides the key components of a robot-assisted minimally invasive surgery system, including patient-side manipulators and surgeon interface elements, while allowing researchers to develop and experimentally validate novel sensing, force estimation, rendering, and control algorithms. Unlike a turn-key clinical system, the dVRK serves as a flexible research platform in which investigators can integrate custom sensing, control, and safety modules according to the objectives of their studies [56]. Because integrating sterilizable, miniaturized multi-axis force sensing directly at the surgical instrument tip remains challenging, many dVRK haptics studies rely on sensorless force estimation (e.g., from motor currents and robot dynamics) and/or external sensing in benchtop phantoms, then render the estimated interaction forces at the surgeon console [45].

A representative user study on dVRK-based haptic feedback reported that force feedback produced a statistically significant benefit in palpation and incision tasks, while the effect was less pronounced for grasping and suturing, highlighting that the value of haptics is task-dependent (strongest when tissue property discrimination or controlled tissue interaction is required) [57].

More recently, sensorless haptic feedback has been shown to yield sizeable task gains in dVRK-style telesurgery experiments: tumor detection accuracy during palpation increased by roughly 30% compared with unilateral (vision-only) teleoperation, and unintended contact forces during a peg-transfer task were reduced by approximately three-fold [45].

Overall, dVRK case studies illustrate a pragmatic pathway to introduce haptics into surgical teleoperation: (i) select a force-sensing/estimation strategy compatible with instrument constraints, (ii) apply filtering/scaling appropriate to human perception and safety limits, and (iii) ensure stability under delay and discretization through conservative gain design and stability-aware control.

#### 5.1.3. Shared Control and Virtual Fixtures

Beyond direct force reflection, shared-control paradigms overlay virtual fixtures, software-defined constraints that guide motion within safe boundaries. In practice, these fixtures act as “virtual rails” that restrict tool motion to planned surgical paths while providing resistive feedback if the operator deviates [36]. This has been applied in orthopedic drilling, ophthalmic microsurgery, and vascular catheter navigation, where millimeter-level accuracy is required. Human–machine collaboration of this kind reduces cognitive load and enhances repeatability, blending surgeon intuition with robotic precision. This was also applied in research for flexible needle insertion aiming for applying the concept in ablation and biopsy and it has shown that vibrotactile feedback for guidance improved the steering accuracy [58,59]. Shared control has also been applied for endoscopic experiments using various haptic feedback methods including kinesthetic and tactile feedback in a study for controlling a soft robotic endoscopic module [60].

#### 5.1.4. Teleoperation and Remote Surgery

Teleoperation enables clinicians to perform procedures remotely by manipulating a follower robot through a master interface while maintaining control over tool–tissue interactions. This capability is particularly attractive in settings where direct access is difficult or hazardous, including military medicine, disaster response, isolation units, and future space-based healthcare. When haptic feedback is incorporated, the teleoperation loop is closed by returning kinesthetic (force/torque) and/or tactile information to the operator, thereby improving situational awareness during tasks such as dissection, palpation, and needle insertion.

A major challenge in haptic teleoperation is the communication channel. Latency, jitter, and packet loss degrade transparency and can destabilize bilateral force feedback, motivating the use of passivity-preserving architectures and delay-robust control strategies (Section 3.4) [18]. Human-factor studies further indicate that telesurgical performance deteriorates as communication delay increases; for example, controlled experiments suggest that delays of approximately 100 ms or less are desirable for maintaining acceptable performance in telesurgery-like tasks [61].

Research and commercial systems illustrate different approaches to haptics-enabled teleoperation. The DLR MiroSurge platform was specifically developed for endoscopic telesurgery with force feedback and supports distal sensing and bilateral control architectures suitable for haptic interaction [62]. In contrast, the clinically deployed Senhance Surgical System incorporates haptic sensing capabilities that return interaction forces to the surgeon, providing a notable example of force feedback in a commercial robotic-assisted surgery platform [63].

Clinical experience with haptics-enabled surgical systems remains relatively limited compared with the large body of experimental research. Nevertheless, emerging clinical reports suggest that force-feedback-assisted robotic surgery is feasible and safe in selected applications. For example, the Senhance Surgical System has been used clinically in pediatric surgery, where observational studies have reported favorable surgical outcomes and demonstrated the practical integration of haptic feedback into routine robotic procedures [64].

Recent developments in Asia further demonstrate the growing clinical interest in haptic robotic surgery. The Saroa surgical robot developed by Riverfield (Tokyo, Japan) incorporates force-feedback functionality and has been successfully used in thoracic surgery, representing one of the first reported clinical applications of a haptic surgical robot [65]. More recently, an initial clinical case series of robot-assisted hysterectomy using the Saroa system further evaluated the practical use of haptic feedback during surgery and highlighted its potential role in improving surgeon perception and control [66]. Other contemporary systems, such as the Versius platform, emphasize modularity, ergonomics, and workflow integration; however, publicly available documentation does not indicate the presence of true force-reflecting haptic feedback, reflecting the broader trend that many commercial surgical robots still rely primarily on visual feedback despite ongoing advances in haptic technology [67].

Together, these examples highlight the diversity of teleoperation architectures and illustrate the continuing challenge of balancing transparency, stability, communication constraints, and clinical usability in haptics-enabled remote surgery.

#### 5.1.5. Haptics in Interventional Robotics

Interventional robotic systems represent one of the most active areas for the clinical translation of haptic technology. In cardiovascular and neurovascular procedures, operators manipulate long, flexible catheters and guidewires while relying primarily on fluoroscopic imaging, resulting in limited perception of vessel-wall interaction forces. The absence of tactile information can increase the risk of excessive contact forces, vessel injury, and navigation errors, making force feedback and haptic guidance particularly valuable.

Recent research has focused on restoring force perception during catheter manipulation. Shi et al. developed a haptic robot-assisted catheter operating system with integrated force feedback and collision protection capabilities to improve operator awareness and navigation safety during vascular interventions [68]. Similarly, Song et al. proposed a passive haptic interface for robot-assisted endovascular catheterization, demonstrating how force feedback can be incorporated into catheter-based teleoperation without compromising system stability [69]. Beyond force reflection, shared-control approaches are increasingly being explored. Qin et al. introduced a haptic shared-control framework based on control barrier functions that combines operator intent with safety constraints, enabling interaction-force regulation while preserving user authority during teleoperation [70].

The field is also moving toward clinically relevant validation. Dagnino et al. reported in-vivo validation of a robotic platform for endovascular intervention, demonstrating the feasibility of teleoperated vascular procedures in realistic biological environments [71]. More recently, Zhang et al. presented an advanced robotic system incorporating haptic feedback for precision cardiac ablation procedures, highlighting the growing role of force feedback in improving procedural safety and accuracy in interventional cardiology [72]. Collectively, these developments illustrate why interventional robotics is increasingly regarded as one of the most promising application domains for medical haptics and exemplify the broader shift from pure force reflection toward information-rich feedback that supports navigation, safety, and decision making.

### 5.2. Rehabilitation and Diagnostic Systems

#### 5.2.1. Haptics in Motor Rehabilitation

Rehabilitation robots use controlled forces and torques to guide, assist, resist, or perturb patient movement after neurological or orthopedic injury. Common interaction paradigms include assist-as-needed support, error augmentation, and impedance-based guidance, all of which exploit haptic interaction to facilitate motor learning and recovery [16].

Evidence from systematic reviews indicates that robot-assisted rehabilitation can improve functional outcomes after stroke when combined with conventional therapy and delivered at sufficient intensity. However, outcomes depend strongly on patient characteristics, intervention dose, and control strategy, highlighting the need to match haptic interaction paradigms to specific therapeutic goals.

Recent evidence further supports the clinical relevance of haptic rehabilitation technologies. Gomez-Risquet et al. reported positive effects of haptic feedback interventions on gait and balance outcomes in post-stroke rehabilitation, highlighting the potential of haptic cueing to enhance motor recovery and functional performance [73]. These findings reinforce the growing view that haptic feedback should be considered not only as a technological enhancement but also as a clinically meaningful component of rehabilitation interventions.

A variety of haptic rehabilitation platforms have been developed to address different therapeutic goals. End-effector robots, such as the MIT-Manus/InMotion family, provide task-space force guidance and assistance for repetitive upper-limb training and motor recovery after stroke [17]. In contrast, upper-limb exoskeletons, including Armeo-class systems such as ArmeoPower, deliver joint-level assistance, resistance, and gravity compensation to support coordinated multi-joint movements during rehabilitation [74]. At the distal level, compact hand and wrist rehabilitation devices focus on restoring dexterity through controlled grip forces and pronation–supination training, with systems such as CR2-Haptic representing efforts toward portable and accessible rehabilitation technologies [54]. Dawson-Elli and Adamczyk developed a validated lower-limb haptic rehabilitation robot for assessment and motor training applications [75]. Although these systems differ substantially in mechanical configuration and target function, they share the common objective of using haptic interaction to increase training intensity, provide objective performance assessment, and support adaptive therapy tailored to individual patient needs. Beyond upper-limb rehabilitation, haptic interaction in a broad sense is increasingly incorporated into lower-limb robotic rehabilitation and repetitive gait training, where the primary objective is typically power assistance, body-weight support, and trajectory guidance rather than high-fidelity force reflection. Unlike surgical applications, these systems commonly employ adaptive impedance, assist-as-needed control, and patient-specific force modulation to accommodate individual movement patterns, muscle weakness, and recovery progression. Furthermore, lower-limb rehabilitation robots generally require substantially higher actuator torques than upper-limb or surgical haptic systems because they must support or assist body-weight-bearing movements during gait. Consequently, haptic controllers are designed not only to reproduce interaction forces where appropriate but also to facilitate motor learning, improve gait symmetry, and promote functional recovery through repetitive, task-oriented training.

#### 5.2.2. Haptic Biofeedback and Objective Assessment

Rehabilitation robots inherently measure and apply interaction forces; this enables quantitative assessment and closed-loop biofeedback beyond what is feasible in manual therapy. Typical measurable variables include interaction force/torque profiles, trajectory error, movement smoothness, timing/coordination metrics, and compliance-related measures. These metrics can support clinicians by (i) tracking recovery longitudinally, and (ii) adjusting therapy difficulty adaptively (e.g., reducing assistance as patient effort increases) [16].

Haptic biofeedback can be delivered in several ways:

- Continuous guidance/resistance fields (e.g., compliant “virtual fixtures” for rehabilitation tasks),
- Event-based cues (e.g., vibrotactile alerts for excessive effort or poor posture),
- Task-embedded haptic rendering in game-like therapy exercises (virtual objects, boundaries, or targets).

A parallel trend is the move toward minimally supervised or home-oriented therapy concepts that combine a haptic device with a structured user interface and exercise design. For example, platform-oriented work built around the ReHapticKnob concept has been reported in the context of enabling robot-assisted, minimally supervised hand therapy workflows [54].

#### 5.2.3. Diagnostic and Palpation Robotics

Beyond therapy, haptic feedback also supports diagnostic interaction. Clinical diagnosis still relies heavily on mechanical interaction, most notably palpation, where clinicians infer abnormalities from changes in stiffness, lumpiness, boundary transitions, or contact events. Robotic palpation and diagnostic manipulation aim to standardize contact, measure forces more reproducibly, and (when appropriate) return haptic cues to the operator for improved perception.

In practice, diagnostic haptics is commonly framed as reliable stiffness/impedance perception under non-ideal conditions (limited bandwidth, noise, and delays). Classic work in soft-tissue telemanipulation shows that enhancing or shaping feedback can improve stiffness perception reliability even when perfect transparency is not achievable, provided feedback remains stable and preserves relative cues [25]. This perspective motivates diagnostic systems that emphasize consistent, interpretable mechanical cues rather than attempting to perfectly reproduce every high-frequency tactile detail.

### 5.3. Training, Simulation, and Prosthetics

#### 5.3.1. Surgical Training and Simulation

Haptic-enabled simulators play a central role in modern medical education. Systems such as LapSim (Surgical Science AB, Gothenburg, Sweden), VirtaMed (VirtaMed AG, Schlieren, Zurich, Switzerland), and Simendo (Simendo B.V., Rotterdam, The Netherlands) combine 3D visualization with kinesthetic devices (Omega.7, Force Dimension SA, Nyon, Switzerland; PHANTOM Premium, 3D Systems Inc., Rock Hill, SC, USA) to recreate realistic tissue interaction. Force feedback helps trainees learn delicate motor control, for instance, identifying the transition from fascia to muscle or judging suture tension [53].

Randomized studies and systematic reviews indicate that adding haptic (force-feedback) to VR surgical simulators can shorten the learning curve and reduce time to proficiency/task completion, particularly for novice trainees, although effects vary by task and platform. However, evidence that haptic-enabled simulation specifically improves outcomes in first real procedures remains limited; most operating-room (OR) transfer evidence relates to simulation training more broadly rather than isolating the incremental contribution of haptic feedback [3].

Haptic simulation has become an important component of modern dental education, where virtual reality simulators enable students to develop fine motor skills, cavity preparation techniques, and tactile perception before treating patients. Commercial and research systems have demonstrated improvements in procedural accuracy, confidence, and student acceptance across multiple stages of dental training. A representative commercial platform is the Simodont^®^ Dental Trainer (Nissin Dental Products Europe B.V., Nieuw-Vennep, The Netherlands), which combines high-fidelity haptic rendering with virtual reality to provide realistic simulation of dental procedures. Recent studies have shown that Simodont is effective for endodontic access cavity training, complements conventional phantom-head training, and supports skill development throughout the dental curriculum, demonstrating high levels of educational acceptance and reinforcing the growing role of haptic technologies in dental education [76,77,78].

#### 5.3.2. Haptic Feedback in Prosthetics

In prosthetic limbs, haptics restores tactile and proprioceptive information to amputees, enabling more natural and confident control. There are three main modalities:

1. Vibrotactile feedback: small vibration motors convey contact events or grip strength to residual skin.
2. Electrotactile feedback: electrical stimulation of skin nerves simulates touch; intensity scales with grip force.
3. Mechanotactile feedback: motor-driven pads apply pressure proportional to object stiffness.

Recent neuro-haptic interfaces employing implanted peripheral nerve electrodes enable upper-limb amputees to receive discriminable tactile and proprioceptive percepts (e.g., via Utah Slanted Electrode Arrays (USEAs) in residual nerves). These systems have shown stable sensory feedback and improved prosthetic control, and users often report enhanced embodiment of the prosthetic limb [79,80].

#### 5.3.3. Assistive and Wearable Haptics

Wearable haptic devices extend assistance to daily living and tele-rehabilitation. Soft gloves using pneumatic actuators provide grip assistance to elderly users, while integrated vibrotactile cues guide hand motion. Smart textiles embedding strain sensors and vibration nodes allow continuous monitoring of muscle engagement during physical therapy [55].

## 6. Challenges and Research Frontiers

Despite significant progress in haptics, clinical translation remains limited because medical use imposes simultaneous constraints on fidelity, stability, integration, usability, and regulatory validation. This section summarizes the most persistent barriers and the research directions most directly addressing them.

### 6.1. Technical Challenges

#### 6.1.1. Rendering Fidelity, Bandwidth, and Device “Z-Width”

The perceived realism of a haptic interaction is ultimately bounded by the combined limitations of the mechatronic device, the sensing chain, and the discrete-time control loop. A useful way to frame these limitations in review papers is to separate (i) what the mechanism can physically do, (ii) what can be measured reliably, and (iii) what can be rendered stably in real time.

From the hardware perspective, the dominant constraint is the device intrinsic mechanical impedance—inertia, viscous/Coulomb friction, transmission compliance, and backlash. High intrinsic impedance degrades the “free-space” feel (users sense device dynamics even when no force should be rendered) and masks small interaction cues that are clinically relevant in delicate manipulation. These effects become more pronounced when the user makes rapid exploratory motions, because inertia and friction scale the effort needed to move the interface and can distort the apparent stiffness of rendered tissues.

Sensing limits further restrict fidelity. Force/torque sensors introduce noise floors, drift, and bandwidth constraints; position and velocity estimation add quantization and filtering delay. In practice, these non-idealities create a trade-off between responsiveness and smoothness: aggressive filtering reduces noise but adds phase lag, while minimal filtering preserves timing but can excite oscillations when coupled with high feedback gains.

Discrete-time implementation is the third major limiter. Sampling, computation, and actuator dynamics can inject “virtual energy” into the loop, reducing the maximum stably renderable stiffness and causing buzzing or limit-cycle oscillations during contact. These effects are often discussed through the lens of the device’s renderable impedance range. In the haptics literature, the term Z-width is commonly used to describe the dynamic range of passively renderable impedances (from near-zero impedance in free space to high impedance during rigid contact) [81,82]. Importantly, Z-width is not only a controller property: it depends strongly on mechanical damping/inertia and on discretization effects, which is why approaches that add (physical or virtual) dissipation can expand the stable range of renderable impedances [82]. A closely related stability-oriented discussion of discretization, quantization, delay, and friction effects on haptic rendering can be found in the teleoperation/haptic-rendering literature [27,83].

#### 6.1.2. Sensing, Miniaturization, and Clinical Integration

Clinical deployment imposes packaging, sterilization, and reliability constraints that are substantially stricter than in laboratory setups. In minimally invasive surgery, the small diameter of instruments makes it difficult to integrate multi-axis force/torque sensors near the tool tip without compromising strength, sterilizability, or cost. Consequently, many prototypes demonstrate excellent haptic performance in benchtop settings but do not translate directly into sterilizable, disposable, or serviceable instruments.

This integration bottleneck has driven two pragmatic directions in recent work. The first is proximal or embedded sensing combined with model-based compensation (e.g., estimating distal contact forces from joint torques, motor currents, and robot dynamics). The second is sensorless or vision-based estimation, where interaction forces are inferred from tool motion and observed tissue deformation. These approaches can provide informative feedback even when direct distal sensing is infeasible, although they introduce their own uncertainty and calibration challenges [45,84,85]. This practical reality is one of the reasons many clinically deployed surgical robots still emphasize visual cues and conservative force/constraint strategies rather than full, high-gain force reflection [21].

#### 6.1.3. Latency and Stability in Networked Teleoperation

In networked teleoperation, transparency is constrained not only by device mechanics but also by communication impairments: delay, jitter, and packet loss reduce phase margin and can destabilize high-gain bilateral control. For this reason, stability-preserving architectures are central to medical-grade designs, particularly when the environment dynamics are uncertain and the human operator cannot be assumed to behave as a fixed linear impedance.

Passivity-based methods are widely used because they provide a conservative but practical safety argument: if the overall interaction remains passive at the human port, stable coupling with typical users is more likely. Time-domain passivity observers/controllers (PO/PC) regulate energy flow by injecting dissipation only when needed, thereby attempting to preserve transparency when interaction is benign while preventing energy generation under adverse conditions [27]. For delayed communication channels, wave/scattering transformations are a well-established approach to preserve passivity of the channel and maintain stability under delay [18]. In the medical context, these methods are often paired with task-dependent compromises: when delays increase, systems may reduce reflected stiffness, filter force signals more aggressively, or switch to informative event-based cues to retain usability while maintaining robust stability.

Finally, the control/communication design must be compatible with human performance limits. Empirical telesurgery-like studies consistently show that increasing delay degrades performance in a task-dependent way, motivating co-design of rendering, prediction/compensation, and user feedback rather than treating latency as a purely technical parameter [61].

### 6.2. Human-Factor Challenges

#### 6.2.1. Perceptual Utility and Calibration

In medical robotics, the goal of haptic feedback is not to maximize physical fidelity in an abstract sense, but to deliver perceptually useful information that supports the clinical task. Human psychophysics imposes practical limits on what users can reliably discriminate, and these limits depend on contact conditions (normal vs. shear), the reference force/stiffness level, exploration strategy (active vs. passive), and user expertise. Consequently, haptic cue design is typically most effective when it prioritizes features that exceed perceptual thresholds (e.g., contact onset/offset, puncture events, relative stiffness differences, or slip-related cues) rather than attempting to recreate every fine-grained interaction detail. A common approach is to calibrate force/stiffness scaling and filtering to stay above just-noticeable-difference (JND) levels for the relevant operating range, while avoiding excessive amplification that increases noise and destabilization risk [14,23,24].

Calibration is also a human-variability problem. Inter-user differences (tactile sensitivity, motor strategy, fatigue) and contextual effects (glove thickness, posture, tool leverage) can shift perceived intensity and interpretation of the same physical stimulus. For review-oriented reporting, it is therefore useful to explicitly state (i) the intended perceptual cue(s), (ii) the mapping and scaling used to encode these cues, and (iii) any user- or task-dependent tuning procedures. This makes results comparable across studies and clarifies whether performance gains are attributable to the feedback channel itself or to a particular encoding design.

#### 6.2.2. Cognitive Load and Fatigue

Haptic feedback can reduce reliance on vision by transferring some interaction monitoring to the tactile/kinesthetic channel, which is consistent with multiple-resource accounts of attention that predict lower interference when information is distributed across modalities [86]. In practice, however, this benefit is strongly contingent on cue quality. If haptic signals are noisy, inconsistent, poorly synchronized with vision, or too intense, they may increase workload by forcing users to resolve cross-modal conflicts or to actively suppress distracting sensations. For this reason, many studies complement performance outcomes (time, error, peak force) with workload instruments such as the NASA Task Load Index (NASA-TLX) to quantify whether a feedback design improves efficiency without imposing additional mental demand [87].

Fatigue is similarly context-dependent. Kinesthetic masters with higher apparent inertia or aggressive stabilizing damping can cause arm/hand fatigue in prolonged procedures, while wearable cutaneous arrays may produce discomfort or sensory adaptation if continuously driven at high intensities. Review-style discussions should therefore connect “haptic benefit” to duration and ergonomics: what improves short benchtop tasks may not translate to long clinical sessions unless the feedback is designed for comfort, low impedance, and minimal attentional overhead. Evidence from multimodal feedback studies supports the general principle that haptic cues should be sparse, task-relevant, and perceptually interpretable to avoid overloading the operator [33].

#### 6.2.3. Evaluation and Standardization Gaps

A persistent translational barrier is the lack of universally accepted, task-independent metrics for “haptic quality” in medical settings. Most evaluations therefore combine (i) system-level measures (delay, bandwidth, force-tracking error, energy/passivity indicators, and stability margins) with (ii) task-level outcomes (accuracy, completion time, safety-relevant force metrics) and (iii) human outcomes (workload, comfort, realism ratings). This mixed-method approach is appropriate, but cross-study comparability remains limited when reporting is incomplete or when experimental tasks differ substantially. For medical systems in particular, aligning evaluation practice with human-centered design and usability engineering frameworks can improve reproducibility: ISO 9241-210 formalizes human-centered design activities for interactive systems, while IEC 62366-1 specifies usability engineering expectations for medical devices [88,89]. Regulatory guidance also emphasizes the role of human factors/usability engineering evidence in demonstrating safe and effective use, motivating more systematic reporting of user populations, use scenarios, and risk-critical tasks [90].

For rehabilitation and wearable systems, broader standardization efforts exist (e.g., ASTM committees for exoskeletons/exosuits), but haptics-specific benchmarking is still fragmented across subfields [91]. As a practical recommendation for review papers, it is helpful to summarize each study with a consistent template (device + cue design, latency/update rate, stability strategy, user/task, primary outcomes, and failure modes), since this makes methodological differences explicit and facilitates meaningful synthesis.

### 6.3. Safety, Reliability, and Regulatory Translation

Haptic medical robots are safety-critical interactive systems: they exchange power with a human operator and (directly or indirectly) influence forces applied to the patient. As a result, safety arguments must address not only mechanical hazards (over-force, unintended motion) but also control-software hazards (instability, oscillations, unexpected transients) that can emerge when feedback gains are increased or when sensing/communication is degraded. From a risk-management perspective, development is typically structured around hazard identification, risk estimation, and risk control with verification of effectiveness, consistent with ISO 14971 for medical-device risk management [92]. At the organizational level, quality management requirements for medical devices are commonly addressed via ISO 13485, which frames design controls, traceability, and lifecycle processes that support regulatory submissions [48].

Because many haptic subsystems are implemented in software (rendering, filtering, stability enforcement, fault handling), software lifecycle rigor becomes central to translation. IEC 62304 defines processes and activities for medical device software development and maintenance, which is especially relevant when haptic controllers include safety monitors, watchdog logic, or state-machine transitions into “safe modes” under abnormal conditions [93]. For systems that fall under medical electrical equipment, IEC 60601-1 provides baseline requirements for basic safety and essential performance, motivating conservative approaches to fault handling and limits on hazardous outputs [49]. In parallel, usability engineering is not optional: the safety case must consider use errors and predictable misuse, which is why IEC 62366-1 and regulatory human-factors guidance are commonly used to structure validation around realistic use scenarios and risk-critical tasks [89,90]. ISO 9241-210 further supports this by formalizing human-centered design activities, helping ensure that feedback encodings are interpretable and do not introduce new cognitive hazards [88].

In practical haptic implementations, safety is usually enforced through layered controls. At the control level, stability-preserving methods (e.g., passivity-based monitoring and energy dissipation, conservative gain scheduling, and delay-aware architectures in teleoperation) are used to prevent energy injection that can lead to oscillations or limit cycles, particularly under sampling and communication delay [18,27]. At the system level, force/velocity saturation, rate limits, and software interlocks reduce the severity of faults and bound worst-case outputs. At the human-interface level, clear warning cues and predictable fallback behavior (e.g., graceful degradation from force reflection to vibrotactile alerts or to visual force overlays) can maintain usability while reducing risk when high-fidelity reflection is unsafe.

For review synthesis, it is useful to explicitly distinguish laboratory feasibility from clinical readiness. Many prototypes demonstrate performance gains under controlled conditions, but clinical translation additionally requires evidence of reliability (repeatability, drift tolerance, sterilization compatibility), comprehensive fault analysis, usability validation with representative users, and traceable verification/validation aligned with the relevant standards and guidance cited above [48,49,89,90,92,93]. This framing clarifies why conservative haptic implementations may deliver smaller performance gains than experimental high-gain force reflection, yet still represent a more realistic pathway to certification and deployment.

## 7. Future Directions in Medical Haptics

The challenges outlined in Section 6 motivate several converging research directions. Future medical haptics is trending from “force reflection whenever possible” toward task-aware, adaptive, and clinically deployable haptic information delivery, including cases where forces are estimated, simplified, or encoded rather than directly measured and replayed.

### 7.1. Data-Driven Haptics and Sensorless Force Estimation

A major direction is replacing fragile or hard-to-integrate distal sensors with model-based and learning-based force estimation from robot signals and/or video. This includes neural-network approaches for tool–tissue force estimation in robot-assisted surgery [84], as well as optical/vision-based estimation strategies that infer interaction forces from observed deformation [85]. Recent telesurgery work also demonstrates that sensorless haptic feedback can deliver measurable task benefits in palpation-style tasks [45]. In parallel, data-driven models are increasingly used for robust prediction of interaction forces in robotic surgery contexts [44].

Practical implication: the most clinically relevant near-term gains may come from reliable, sterilizable force inference coupled with conservative stability-aware rendering, rather than perfect direct sensing everywhere.

### 7.2. Emerging Interface Technologies

#### 7.2.1. Multimodal and XR-Enabled Guidance

Haptics is increasingly combined with augmented reality (AR), virtual reality (VR), and mixed reality (MR) to provide complementary channels: vision for spatial context and haptics for constraint/interaction cues. Studies combining augmented reality with haptics for surgical planning and interaction indicate that multimodal designs can improve usability and clarity when visual overlays and force cues are aligned [94]. The design priority is not maximal feedback, but well-synchronized, interpretable cues that reduce uncertainty without adding workload [33].

#### 7.2.2. Soft, Wearable, and Home-Compatible Interfaces

Soft robotics and wearable actuation are enabling haptic systems that are lighter, safer, and more compatible with rehabilitation and home use. Soft, compliant devices reduce risk during physical interaction and can improve comfort in long-duration sessions—an important factor for rehabilitation and assistive care [19].

#### 7.2.3. Neurohaptics and Bidirectional Human–Robot Interfaces

Neurohaptics seeks to restore or augment touch perception by establishing direct communication between robotic systems and the peripheral or central nervous system through implanted or non-invasive neural interfaces. Sensory information acquired by robotic sensors can be conveyed via peripheral nerve stimulation, intraneural electrodes, or cortical stimulation, while neural recordings can be used to decode motor intention, enabling closed-loop human–robot interaction. Recent studies have demonstrated stable tactile percepts through neural interfaces [79], discriminable artificial texture sensations using intraneural stimulation in both intact and amputee users [95], and the first clinical implementation of bidirectional somatosensory neuroprosthetic systems [96]. Although these technologies remain at an early stage of clinical translation, challenges related to long-term implantation stability, safety, and ethical considerations must be addressed before widespread clinical adoption. Nevertheless, neurohaptics represents a promising direction toward intuitive sensory restoration and advanced prosthetic and assistive systems.

#### 7.2.4. Networked Haptics and the “Internet of Skills”

Networked haptics aims to share tactile expertise across distance. Concepts such as the “tactile internet” propose ultra-low latency and high reliability targets for haptic communication, which—if achievable in medical-grade systems—could expand remote mentoring, training, and collaboration [97]. From a practical standpoint, these systems must be designed around both control stability (Section 3) and human delay tolerance [61].

### 7.3. Outlook and Research Priorities

Future progress in medical haptics is likely to be driven less by maximizing physical realism and more by task-oriented haptic information design: delivering clinically relevant cues in a form that is safe, interpretable, and compatible with clinical workflow. In many procedures, the most valuable information is not the complete tool–tissue interaction spectrum, but specific cues such as contact onset, tissue transitions, excessive force, slip, proximity to sensitive anatomy, or impending constraint violations. This motivates haptic systems that prioritize utility, stability, and ergonomics over raw fidelity [4,13,14].

A particularly promising direction is the use of guidance haptics in surgery. Virtual fixtures, guidance fields, and shared-control strategies can encode safe trajectories, keep-out regions, and force boundaries, improving precision and reducing errors without requiring full force reflection [35,36,60]. Recent developments in shared-control frameworks for robotic intervention, including control-barrier-function-based haptic guidance, further demonstrate how safety constraints and operator intent can be integrated through haptic interaction rather than relying solely on direct force reflection [70]. Closely related is the concept of sensory augmentation, where feedback is intentionally amplified or reshaped to highlight clinically meaningful events such as contact with hard tissue, proximity to vulnerable anatomy, or subtle stiffness transitions. Such information-centric feedback can improve situational awareness and support safer manipulation, provided that amplification remains perceptually meaningful and does not compromise stability.

Another important research priority is the development of feedback modalities that preserve operator comfort and freedom of movement. High-authority kinesthetic feedback can increase apparent inertia and constrain motion, while continuous vibrotactile or thermal stimulation may cause discomfort, sensory adaptation, or distraction. Consequently, there is growing interest in lightweight cutaneous interfaces, contactless mid-air haptics, and adaptive cue scheduling that delivers feedback only when clinically relevant [9,20]. Event-based and user-adaptive feedback strategies may offer a better balance between information delivery, comfort, and long-term usability.

Recent developments in wearable rehabilitation haptics further illustrate the trend toward lightweight and home-compatible systems. Examples include tension-driven upper-limb rehabilitation robots [41], soft robotic gloves with integrated sensing and force feedback for virtual-reality-based rehabilitation [40], and earlier haptic rehabilitation systems that combined robotic assistance with interactive therapeutic gaming environments [98]. Together, these technologies demonstrate the feasibility of delivering intensive, task-oriented, and personalized rehabilitation through compact haptic interfaces outside conventional clinical settings. Although many of these systems remain at the prototype stage, early user studies have reported encouraging results in terms of engagement, task performance, and therapy delivery. Nevertheless, most evaluations have been limited to pilot studies or small user cohorts, and larger clinical trials are still required to establish long-term therapeutic efficacy, usability, and clinical adoption.

Across all approaches, robust sensorless or indirect force estimation remains a key enabling technology wherever distal force sensing is impractical [45,84]. Equally important is the development of evaluation frameworks that relate haptic design choices to clinically meaningful outcomes, including force safety, targeting accuracy, workload, fatigue, and error recovery [1,14]. Together, these directions support a shift toward clinically deployable haptic systems that emphasize guidance, awareness, safety, and usability while maintaining the stability and reliability required for medical practice.

## 8. Conclusions

Haptics has matured from a niche enabling technology into a central design consideration for safe and effective medical robotics. Across surgery, rehabilitation, simulation, and prosthetics, the recurring message is that touch information—whether rendered as force reflection, cutaneous cues, or guidance fields—can improve interaction quality when it is stable, interpretable, and aligned with clinical workflow. Rather than treating haptics as a single feature, the literature supports viewing it as a system-level capability that depends jointly on sensing/estimation, rendering and control, human factors, and integration constraints.

### 8.1. Synthesis of Evidence and Key Takeaways

First, medical haptics is inherently multichannel. Human perception integrates cutaneous and kinesthetic information, and effective interfaces often combine these channels to provide complementary cues (e.g., event detection and slip cues via cutaneous feedback alongside gross force/impedance cues via kinesthetic feedback). This motivates multimodal designs and, importantly, cue selection: many clinical tasks benefit more from a small set of well-timed cues (contact onset, stiffness transition, excessive force, proximity to sensitive anatomy) than from attempting to reproduce the full interaction spectrum.

Second, stability and safety dominate haptic rendering in medical contexts. The transparency–stability trade-off is not merely a theoretical concern; it directly shapes the realizable stiffness, damping, and bandwidth under discrete-time control, device dynamics, and (in teleoperation) communication delay. Accordingly, stability-preserving methods (passivity-based approaches, conservative gain design, energy shaping, and delay-robust architectures) remain central to any credible path toward clinical deployment.

Third, the evidence base indicates that haptic feedback can improve performance, but the magnitude and generality of the benefit depend on the task, user expertise, and how feedback is encoded. Meta-analyses and systematic reviews consistently report reduced applied forces and improvements in time/accuracy in many surgical and simulation tasks, with especially clear utility in tasks requiring careful force regulation and tissue property discrimination. In rehabilitation, haptic interaction is tightly coupled to therapy paradigms (assist-as-needed, error augmentation, impedance guidance), and benefits depend strongly on dose, population, and integration into clinical protocols.

Fourth, translation barriers remain coupled rather than singular. The limiting factors are rarely only actuator strength or sensor resolution; they are often the combination of distal sensing constraints (sterilization, size), limited or noisy force signals, stability margins under delay, user comfort and acceptance, and the regulatory requirements for risk management, verification, and usability engineering. These constraints explain why many commercial surgical systems still do not provide high-fidelity force reflection and why proxy feedback channels (cutaneous cues, guidance haptics, augmented feedback) are increasingly explored.

Finally, force inference and task-oriented rendering are enabling directions. Sensorless and indirect force estimation (model-based and learning-based) can reduce reliance on hard-to-integrate distal sensors, but must be paired with stability-aware rendering and conservative safety envelopes. Equally important, haptic guidance (virtual fixtures and shared control) and sensory augmentation (reshaping or magnifying cues to highlight safety-relevant events) provide pragmatic clinical value even when absolute force fidelity is limited, because they are fundamentally information-driven.

The evidence synthesized in this review provides the following answers to the research questions posed in Section 1.3: RQ1: tactile, kinesthetic, and hybrid systems each occupy distinct but complementary roles; RQ2: safe rendering depends on balancing transparency and stability through passivity-aware control; RQ3: haptic feedback improves performance in many surgical, rehabilitation, and training tasks; RQ4: sensing, integration, human factors, and regulatory requirements remain the principal barriers to adoption; and RQ5: an information-centric perspective provides a useful framework for comparing technologies and identifying clinically meaningful design objectives.

### 8.2. Clinical and Design Implications

For surgical systems, the most clinically actionable haptic opportunities often lie in guidance and awareness rather than full force reflection. Guidance haptics (virtual fixtures, keep-out zones, alignment assistance) can support targeting and precision while directly encoding safety constraints. Sensory augmentation can be used to emphasize transitions (e.g., soft–hard tissue boundaries), proximity to sensitive anatomy, or unsafe force trends, improving situational awareness and potentially reducing trauma. Where force reflection is implemented, it should be designed around safe impedance bounds, predictable scaling, and robust handling of delay and uncertainty.

For rehabilitation, haptic interaction should be matched to the therapeutic objective: assistance and gravity compensation for enabling movement, resistance for strengthening and motor control, and carefully designed perturbations or error augmentation for motor learning. Practical deployment increasingly favors systems that can deliver high-repetition training with minimal supervision, supported by objective metrics and adaptive progression. Comfort, donning/doffing time, and patient tolerance are often as important as raw actuation capability.

For training and simulation, haptics is most valuable when it reinforces decision-relevant cues (contact events, force limits, layer transitions, slip) and when simulator metrics map to clinically meaningful outcomes. High-fidelity rendering is beneficial, but realism should be judged by whether it improves skill acquisition, reduces unsafe force habits, and supports transfer to clinical tasks. This encourages validation designs that report not only face validity and user preference, but also objective force/trajectory metrics and learning curves.

### 8.3. Recommendations for Research and Translation

To strengthen both the scientific foundation and the translational pathway of medical haptics, several priorities emerge:

(1) Task-driven cue design and comparative studies.

It is considered a cornerstone of an information-centric approach, as it prioritizes clinical meaning over raw fidelity. More work is needed to formalize which cues matter for which procedures and to compare cue encodings across modalities (kinesthetic vs. cutaneous vs. hybrid) using consistent outcome measures. This includes principled mappings for guidance and augmentation that preserve interpretability (e.g., monotonic “more cue → higher risk” relationships) while avoiding unnecessary constraint of user motion.

(2) Stability-aware rendering under uncertainty.

Controllers and rendering strategies should be evaluated under realistic conditions: discrete-time effects, actuator saturation, friction/backlash, network delay/jitter, and user variability. Reporting stability margins, safety limits, and failure-handling behaviors alongside performance outcomes would improve reproducibility and reviewer confidence.

(3) Clinically practical sensing and inference.

Since sterilizable distal sensing remains a bottleneck, robust force inference from robot signals and vision should be paired with conservative rendering and safety envelopes. This should include uncertainty-aware approaches (confidence estimation) that modulate feedback strength when estimates degrade.

(4) Human factors as primary design constraints.

Operator freedom, fatigue, adaptation, and acceptability should be treated as first-order requirements. This motivates lightweight and less intrusive displays (cutaneous and contactless approaches), event-based cue scheduling, and personalization of intensity and timing. The goal is feedback that informs without irritating or restricting movement.

(5) Benchmarks that connect engineering metrics to clinical endpoints.

Progress will accelerate if the field converges on shared benchmarks that couple device/control metrics (e.g., impedance range, tracking error, delay tolerance) to clinically interpretable outcomes (applied force safety metrics, precision near anatomy, error recovery, workload, and fatigue). Such benchmarks would also facilitate meta-analysis and help regulators and clinicians interpret the value of haptic features.

This review has several limitations. Although the literature search and study selection followed a systematic methodology, the synthesis was qualitative rather than quantitative because of the methodological heterogeneity of the included studies. Consequently, no formal risk-of-bias assessment or meta-analysis was performed. Nevertheless, the systematic search strategy and structured thematic synthesis provide a comprehensive overview of current advances, challenges, and future directions in medical haptics.

### 8.4. Concluding Perspective

Haptics is unlikely to achieve widespread clinical adoption in medical robotics as a one-size-fits-all pursuit of perfect physical realism. The most durable trajectory is toward information-centric haptics: stable, workflow-compatible feedback that supports guidance, safety, and awareness while respecting the operator’s freedom and comfort. As sensing/estimation, rendering algorithms, and wearable/soft interfaces continue to evolve, haptic feedback can transition from an optional enhancement to a clinically meaningful interaction layer—one that helps clinicians and patients benefit from robotics not only through precision and reach, but also through the restored ability to feel what the robot is doing.

## Figures and Tables

**Table 1:** Literature search domains and representative search queries used across Scopus, PubMed, and IEEE Xplore. The exact query syntax was adapted to the requirements of each database while preserving the same conceptual search domains and inclusion logic.

Search Domain	Representative Search Query
Medical haptics	“haptic feedback” AND “medical robotics”
Surgical force feedback	“force feedback” AND “robotic surgery”
Tactile perception	“tactile perception” AND “medical robotics”
Rehabilitation robotics	rehabilitation AND haptic AND “medical robot”
Teleoperation	teleoperation AND haptic AND “medical robotics”
Prosthetics	prosthetic AND haptic AND “medical robotics”
Surgical simulation	“surgical simulation” AND haptic AND “medical robotics”

**Table 2:** Primary cutaneous mechanoreceptors, their stimulus sensitivities, and functional contributions to human haptic perception.

Receptor Type	Stimulus Sensitivity	Frequency Range	Function
Merkel cells (SA I)	Static pressure, edges	0–10 Hz	Detect texture and shape
Meissner corpuscles (RA I)	Low-frequency vibration	5–50 Hz	Perceive slip and motion onset
Ruffini endings (SA II)	Skin stretch	0–100 Hz	Encode hand conformation
Pacinian corpuscles (RA II)	High-frequency vibration	60–300 Hz	Detect fine texture and impact

**Table 3:** Key characteristics, control variables, and application domains of impedance and admittance control architectures for haptic interaction.

Framework	Measured Variable	Controlled Variable	Best Suited for
Impedance control	Motion ($x,\dot{x}$)	Force (*F*)	Low-inertia, backdrivable interfaces (desktop grounded masters, high-bandwidth devices)
Admittance control	Force (*F*)	Motion/position	Higher-inertia or less backdrivable robots; systems where accurate motion control is easier than accurate force output

**Table 4:** Representative psychophysical and system-level constraints relevant to haptic rendering, adapted from representative studies on force discrimination, stiffness perception, perceptual latency, and surgical tool–tissue interaction forces [14,23,24,31]. JND = just noticeable difference. While psychophysical thresholds primarily reflect properties of human perception and are broadly applicable across medical procedures, tool–tissue interaction forces vary substantially with the clinical context [4,31].

Parameter	Representative Threshold	Design Implication
Force discrimination (kinesthetic JND/Weber fraction)	≈5–10% of reference force	Prioritize stability and noise reduction over force resolution below perceptual discrimination thresholds.
Stiffness discrimination (JND/Weber fraction)	≈20–30% (procedure-dependent)	Target stiffness rendering errors of approximately 20–30% or lower when stiffness perception is task critical.
Perceptual latency (end-to-end haptic delay)	Tens of milliseconds	Minimize end-to-end latency through high-rate control and delay-compensation strategies.
Tool–tissue interaction force range (surgical tasks)	Task-dependent	Size sensors, actuators, and safety limits according to the intended clinical application.

**Table 5:** Comparison of the principal haptic feedback modalities used in medical robotics, highlighting the information conveyed, representative applications, advantages, and limitations of each approach.

Modality	Information Conveyed	Typical Applications	Advantages	Limitations
Kinesthetic (force feedback)	Force magnitude, stiffness, impedance, guidance	Microsurgery, teleoperation, catheter navigation	Natural interaction, high realism	Motion constraint, sensitivity to delay
Cutaneous (skin feedback)	Contact events, slip, warnings, directional cues	Needle guidance, rehabilitation, wearable systems	Lightweight design, low power consumption	Limited force rendering capability
Hybrid (combined)	Force information and localized events	Advanced teleoperation, simulation, prosthetics	High information content, multimodal redundancy	Increased complexity, calibration requirements

**Table 6:** Representative commercial and research haptic platforms used in medical robotics, highlighting feedback modality, implementation status, application domain, strengths, and practical limitations.

System	Feedback Type	Clinical Status	Typical Application	Advantages	Limitations
Virtuose 6D	Kinesthetic	Commercial	Teleoperation, simulation	Large workspace, force/torque rendering	Grounded architecture, high cost
Omega.7/ Sigma.7	Kinesthetic	Commercial	Surgical simulation, research	High bandwidth, high precision	Limited workspace
Touch X	Kinesthetic	Commercial	Training, simulation	Compact design, broad adoption	Limited force authority
Senhance	Kinesthetic force feedback	Clinical deployment	Robot-assisted surgery	Integrated force sensing, clinical deployment	Limited long-term haptic outcome data
Saroa	Kinesthetic force feedback	Early clinical deployment	Thoracic and gynecological surgery	Integrated force feedback, clinical translation	Limited clinical evidence
SenseGlove Nova	Hybrid	Commercial	Training, rehabilitation	Wearable design, multimodal feedback	Limited force rendering capability
Ultraleap	Cutaneous (mid-air)	Commercial	Touchless interaction	Contactless operation, hygienic interface	Limited force magnitude
Simodont^®^ Dental Trainer	Kinesthetic	Commercial	Dental education, simulation	Validated educational platform, realistic force feedback	Training only; not for clinical use

## Data Availability

The literature search data and study selection records supporting the findings of this review are available from the author upon reasonable request.

## Supplementary Materials

The following supporting information can be downloaded at: https://www.mdpi.com/article/10.3390/s26154824/s1, Table S1: Completed PRISMA 2020 Checklist. Reference [99] is cited in the supplementary materials.
