# Supplementary material for: A Systematic Review on Haptic Feedback in Medical Robotics: Technologies, Applications, Clinical Translation, and an Information-Oriented Perspective
Source: Sensors (Basel). 2026 Jul 30;26(15):4824. doi: 10.3390/s26154824 (PMC13468719; doi:10.3390/s26154824)
Supplement: Supplementary file 1 [file sensors-26-04824-s001.zip › sensors-4423280-supplementary.pdf]

PRISMA 2020 Checklist

Manuscript Title:

A Systematic Review on Haptic Feedback in Medical Robotics: Technologies, Applications, Clinical Translation, and an Information-Oriented Perspective

Table S1: Completed PRISMA 2020 Checklist.

Section and Topic	Item	Checklist item	Location where item is reported
**TITLE**			
Title	1	Identify the report as a systematic review.	Title page (Page 1).
**ABSTRACT**			
Abstract	2	See the PRISMA 2020 for Abstracts checklist.	Abstract (Page 1).
**INTRODUCTION**			
Rationale	3	Describe the rationale for the review in the context of existing knowledge.	Section 1.2 (Pages 2–3).
Objectives	4	Provide an explicit statement of the objective(s) or question(s) the review addresses.	Sections 1.2 and 1.3 (Pages 2–4).
**METHODS**			
Eligibility criteria	5	Specify the inclusion and exclusion criteria for the review and how studies were grouped for the syntheses.	Section 1.4 (Pages 4–5).
Information sources	6	Specify all databases, registers, websites, organisations, reference lists, and other sources searched or consulted to identify studies. Specify the date when each source was last searched or consulted.	Section 1.4 and Table 1 (Pages 4–5).
Search strategy	7	Present the full search strategies for all databases, registers, and websites, including any filters and limits used.	Table 1 and Section 1.4 (Page 4).
Selection process	8	Specify the methods used to decide whether a study met the inclusion criteria of the review, including how many reviewers screened each record and each report retrieved, whether they worked independently, and if applicable, details of automation tools used in the process.	Section 1.4 and Figure 1 (Pages 4–6).
Data collection process	9	Specify the methods used to collect data from reports, including how many reviewers collected data from each report, whether they worked independently, any processes for obtaining or confirming data from study investigators, and if applicable, details of automation tools used in the process.	Section 1.4 (Pages 4–5).
Data items	10a	List and define all outcomes for which data were sought. Specify whether all results that were compatible with each outcome domain in each study were sought.	Research Questions (Section 1.3) and Section 1.4.
Data items	10b	List and define all other variables for which data were sought (e.g., participant characteristics, intervention characteristics, publication details). Describe assumptions made about missing information.	Section 1.4 (Pages 4–5).

Section and Topic	Item	Checklist item	Location where item is reported
Study risk of bias assessment	11	Specify the methods used to assess risk of bias in the included studies, including details of the tool(s) used, how many reviewers assessed each study and whether they worked independently, and if applicable, details of automation tools used in the process.	Not applicable. This review presents a qualitative thematic synthesis and did not perform a formal risk-of-bias assessment because of the methodological heterogeneity of the included studies.
Effect measures	12	Specify for each outcome the effect measure(s) (e.g., risk ratio, mean difference) used in the synthesis or presentation of results.	Not applicable. No quantitative synthesis or meta-analysis was performed.
Synthesis methods	13a	Describe the processes used to decide which studies were eligible for each synthesis.	Sections 1.3 and 1.4 (Pages 3–5).
Synthesis methods	13b	Describe any methods required to prepare the data for presentation or synthesis, such as handling of missing summary statistics or data conversions.	Not applicable. Qualitative thematic synthesis only.
Synthesis methods	13c	Describe any methods used to tabulate or visually display results of individual studies and syntheses.	Figures 1–5 and Tables 1–6 throughout the manuscript.
Synthesis methods	13d	Describe any methods used to synthesize results and provide a rationale for the choice(s). If meta-analysis was performed, describe the model(s), methods used to identify statistical heterogeneity, and software package(s).	Sections 1.4 and 8.1. A qualitative thematic synthesis was conducted because of methodological heterogeneity.
Synthesis methods	13e	Describe any methods used to explore possible causes of heterogeneity among study results.	Not applicable.
Synthesis methods	13f	Describe any sensitivity analyses conducted to assess the robustness of the synthesized results.	Not applicable.
Reporting bias assessment	14	Describe any methods used to assess risk of bias due to missing results in a synthesis (arising from reporting biases).	Not applicable. Reporting bias was not formally assessed.
Certainty assessment	15	Describe any methods used to assess certainty (or confidence) in the body of evidence for an outcome.	Not applicable. No GRADE or equivalent certainty assessment was performed.
**RESULTS**			
Study selection	16a	Describe the results of the search and selection process, from the number of records identified to the number of studies included in the review, ideally using a flow diagram.	Section 1.4 and Figure 1 (Pages 4–6).
Study selection	16b	Cite studies that might appear to meet the inclusion criteria but which were excluded, and explain why they were excluded.	Figure 1 and Section 1.4 describe the categories and reasons for exclusion. Individual excluded studies were not listed.

Section and Topic	Item	Checklist item	Location where item is reported
Study characteristics	17	Cite each included study and present its characteristics.	Sections 2–7; representative studies are summarized throughout the text and in Tables 5 and 6.
Risk of bias in studies	18	Present assessments of risk of bias for each included study.	Not applicable. No formal risk-of-bias assessment was performed.
Results of individual studies	19	For all outcomes, present summary statistics and effect estimates for each study, where appropriate.	Representative findings from individual studies are summarized throughout Sections 4–7. Quantitative effect estimates are reported where available from the original studies.
Results of syntheses	20a	For each synthesis, briefly summarise the characteristics and risk of bias among contributing studies.	Sections 4–8 summarize the findings according to application domains (surgery, rehabilitation, prosthetics, simulation, and emerging technologies). Risk of bias was not formally assessed because this review provides a qualitative thematic synthesis.
Results of syntheses	20b	Present results of all statistical syntheses conducted. If meta-analysis was done, present the summary estimate and its precision, and measures of statistical heterogeneity.	Not applicable. No quantitative synthesis or meta-analysis was performed.
Results of syntheses	20c	Present results of all investigations of possible causes of heterogeneity among study results.	Not applicable.
Results of syntheses	20d	Present results of all sensitivity analyses conducted to assess the robustness of the synthesized results.	Not applicable.
Reporting biases	21	Present assessments of risk of bias due to missing results (arising from reporting biases) for each synthesis assessed.	Not applicable. Reporting bias was not formally evaluated.
Certainty of evidence	22	Present assessments of certainty (or confidence) in the body of evidence for each outcome assessed.	Not applicable. Formal certainty assessment (e.g., GRADE) was not performed.
**DISCUSSION**			
Discussion	23a	Provide a general interpretation of the results in the context of other evidence.	Section 8.1 (Synthesis of Evidence and Key Takeaways), Pages 34–35.

Section and Topic	Item	Checklist item	Location where item is reported
Discussion	23b	Discuss any limitations of the evidence included in the review.	Sections 6 and 8.3 discuss limitations of the available literature, including sensing constraints, limited clinical validation, and heterogeneity among studies.
Discussion	23c	Discuss any limitations of the review processes used.	Section 8.3 (Recommendations for Research and Translation), Pages 35–36. The manuscript explicitly states that the review employed qualitative thematic synthesis rather than quantitative meta-analysis because of methodological heterogeneity and therefore did not perform a formal risk-of-bias assessment.
Discussion	23d	Discuss implications of the results for practice, policy, and future research.	Sections 8.2–8.4 (Clinical and Design Implications, Recommendations for Research and Translation, and Concluding Perspective).
**OTHER INFORMATION**			
Registration and protocol	24a	Provide registration information for the review, including register name and registration number, or state that the review was not registered.	Not registered.
Registration and protocol	24b	Indicate where the review protocol can be accessed, or state that a protocol was not prepared.	No review protocol was prepared.
Registration and protocol	24c	Describe and explain any amendments to information provided at registration or in the protocol.	Not applicable.
Support	25	Describe sources of financial or non-financial support for the review, and the role of the funders or sponsors in the review.	Funding and Acknowledgments (Page 36). The review received no external funding. Institutional support from King Fahd University of Petroleum and Minerals (KFUPM) is acknowledged.
Competing interests	26	Declare any competing interests of review authors.	Conflicts of Interest (Page 36). The author declares no competing interests.

Section and Topic	Item	Checklist item	Location where item is reported
Availability of data, code and other materials	27	Report which of the following are publicly available and where they can be found: template data collection forms; data extracted from included studies; data used for all analyses; analytic code; any other materials used in the review.	Data Availability Statement (Page 36). The literature search data and study selection records supporting the findings of this review are available from the corresponding author upon reasonable request.

Notes

- This review follows the PRISMA 2020 reporting recommendations for systematic reviews.
- Because the included studies encompass diverse application domains, methodologies, experimental designs, and outcome measures, a qualitative thematic synthesis was performed rather than a quantitative meta-analysis.
- Consequently, PRISMA items relating specifically to quantitative synthesis, meta-analysis, risk-of-bias assessment, certainty assessment, reporting bias assessment, and sensitivity analyses are reported as "Not applicable".
- The study selection process followed a PRISMA-inspired workflow including systematic database searching, duplicate removal, eligibility screening, full-text assessment, and qualitative synthesis.

This checklist has been prepared according to:

Page MJ, McKenzie JE, Bossuyt PM, Boutron I, Hoffmann TC, Mulrow CD, et al.
The PRISMA 2020 statement: an updated guideline for reporting systematic reviews.
BMJ. 2021;372:n71. doi:10.1136/bmj.n71

This checklist accompanies the manuscript:

A Systematic Review on Haptic Feedback in Medical Robotics: Technologies, Applications, Clinical Translation, and an Information-Oriented Perspective
